# Reading LINEs within the cocaine addicted brain

**DOI:** 10.1002/brb3.678

**Published:** 2017-04-06

**Authors:** Glenn A. Doyle, Tara T. Doucet‐O'Hare, Matthew J. Hammond, Richard C. Crist, Adam D. Ewing, Thomas N. Ferraro, Deborah C. Mash, Haig H. Kazazian, Wade H. Berrettini

**Affiliations:** ^1^ Department of PsychiatryCenter for Neurobiology and BehaviorUniversity of Pennsylvania Perelman School of MedicinePhiladelphiaPAUSA; ^2^ Johns Hopkins School of MedicineInstitute of Genetic MedicineBaltimoreMDUSA; ^3^ Mater Research Institute – University of QueenslandBrisbaneQldAustralia; ^4^ Department of Biomedical SciencesCooper Medical School of Rowan UniversityCamdenNJUSA; ^5^ Department of Neurology, Brain Endowment Bank™University of Miami Miller School of MedicineMiamiFLUSA

**Keywords:** addiction, LINE1, mutation, neuronal development, retrotransposon

## Abstract

**Introduction:**

Long interspersed element (LINE)‐1 (L1) is a type of retrotransposon capable of mobilizing into new genomic locations. Often studied in Mendelian diseases or cancer, L1s may also cause somatic mutation in the developing central nervous system. Recent reports showed L1 transcription was activated in brains of cocaine‐treated mice, and L1 retrotransposition was increased in cocaine‐treated neuronal cell cultures. We hypothesized that the predisposition to cocaine addiction may result from inherited L1s or somatic L1 mobilization in the brain.

**Methods:**

Postmortem medial prefrontal cortex (mPFC) tissue from 30 CA and 30 control individuals was studied. An Alexafluor488‐labeled NeuN antibody and fluorescence activated nuclei sorting were used to separate neuronal from non‐neuronal cell nuclei. L1s and their 3' flanking sequences were amplified from neuronal and non‐neuronal genomic DNA (gDNA) using L1‐seq. L1 DNA libraries from the neuronal gDNA were sequenced on an Illumina HiSeq2000. Sequences aligned to the hg19 human genome build were analyzed for L1 insertions using custom “L1‐seq” bioinformatics programs.

**Results:**

Previously uncataloged L1 insertions, some validated by PCR, were detected in neurons from both CA and control brain samples. Steady‐state L1 mRNA levels in CA and control mPFC were also assessed. Gene ontology and pathway analyses were used to assess relationships between genes putatively disrupted by novel L1s in CA and control individuals. L1 insertions in CA samples were enriched in gene ontologies and pathways previously associated with CA.

**Conclusions:**

We conclude that neurons in the mPFC harbor L1 insertions that have the potential to influence predisposition to CA.

## 1 Introduction

Cocaine addiction (CA) is a debilitating disease affecting 0.5% of the American population, with 1.5 million people (ages 18 and older) having reported using cocaine at least once during the last month (SAMHSA, 2014). Five to six percent of cocaine users will develop CA (O'Brien & Anthony, 2005). CA treatment consists of psychotherapy and self‐help groups, which do not provide benefits for many patients (Alterman, McKay, Mulvaney, & McLellan, 1996; Carroll et al., 2004; Kampman et al., 2001). Improved treatment for CA is needed as dropout rates in treatment programs are high (Kampman et al., 2002), relapse is common among patients who complete treatment (McKay et al., 2010), and mortality among CA individuals is 4–8 fold greater compared to age and sex‐matched peers (Degenhardt et al., 2011). Despite decades of controlled clinical trials, with some medications showing efficacy in preclinical animal models, no FDA‐approved pharmacotherapy exists for CA. Thus, in order to identify novel targets for therapeutic drug development, a more complete characterization of the neurobiology of CA is needed.

Long interspersed element (LINE)‐1 (L1) is a mobile DNA element that constitutes ~17% of the human genome (Lander et al., 2001). Full‐length L1s are ~6 kb long, with a promoter, 5' and 3' untranslated regions, and two open reading frames (ORFs) (Scott et al., 1987). ORF1 encodes an RNA‐binding protein (Martin, 2010) and ORF2 encodes an endonuclease and reverse transcriptase (Feng, Moran, Kazazian, & Boeke, 1996; Mathias, Scott, Kazazian, Boeke, & Gabriel, 1991) that enable L1s to replicate by a “copy‐and‐paste” mechanism to move and accumulate within the genome. Most L1s have lost “genomic mobility” due to truncations or mutations; however, about 100 full‐length L1s in an average human genome, mostly L1Hs *T*_a_ subfamily members, remain “competent” to replicate and insert at a new locus (Richardson et al., 2015).

Kazazian et al. (1988) first demonstrated that a germline L1 retrotransposition event caused human disease. Since then, about 125 germline L1 retrotransposition‐mediated gene disruptions have been shown to cause Mendelian diseases (Hancks & Kazazian, 2016). Somatic mutations by other types of repetitive elements and pseudogenes, dependent upon the L1‐encoded machinery for mobility, have been documented to cause several human diseases (Richardson et al., 2015). Somatic L1 retrotransposition events occur often during embryogenesis (Kano et al., 2009) and in cancerous tissues (Tubio et al., 2014). Numerous studies have shown that L1s can mobilize in both mouse and human brains (Baillie et al., 2011; Evrony et al., 2015; Hazen et al., 2016; Muotri et al., 2005). Greater L1 retrotransposon burdens were reported in DNA from postmortem brains of patients with ataxia telangiectasia (Coufal et al., 2011) and schizophrenia (Bundo et al., 2014).

Given reports of transcriptional activation of L1s in brains of cocaine‐treated mice (Maze et al., 2011) and increased L1 retrotransposition in cocaine‐treated neuronal cell cultures (Okudaira, Ishizaka, & Nishio, 2014), we proposed two hypotheses for how L1 retrotransposons might influence susceptibility to CA, or its symptoms. First, certain inherited (germline L1s) or noninherited (*de novo* somatic L1 retrotransposition during neurogenesis) L1 gene disruptions might predispose individuals to developing CA. Second, by relieving L1 transcriptional repression, epigenetic changes, caused by chronic cocaine‐taking (Maze et al., 2011), create opportunities for increased L1 transcription and *de novo* somatic L1 retrotransposition during adult neurogenesis or in postmitotic neurons (Macia et al., 2017), leading to the cognitive impairments seen in CA individuals (Spronk, van Wel, Ramaekers, & Verkes, 2013).

This is the first *ex vivo* study of human brain L1 retrotransposition events in a drug addiction. We studied medial prefrontal cortex (mPFC) because cocaine's rewarding effects are largely due to blockade of dopamine reuptake at ventral tegmental area nerve terminals, some of which synapse on neurons in the mPFC (Koob & Volkow, 2010). Moreover, reciprocal mPFC glutamatergic corticostriatal neurons are intimately involved in neuroadaptation to cocaine (Kalivas, 2009; Schmidt & Pierce, 2010). We analyzed mPFC from 30 CA individuals who died of cocaine overdose and 30 age, sex, and ethnicity matched control individuals for increased L1 transcription and for genetic L1 burden. We found previously uncataloged L1 insertions in genes within gene ontologies and pathways relevant to cocaine addiction in CA mPFC samples that were absent from control mPFC samples.

## 2 Materials and Methods

### 2.1 Postmortem brain tissue

All subjects were judged by a forensic pathologist to have died of cocaine overdose. Subject demographics, postmortem brain characteristics (postmortem interval before freezing) and DSM‐IV diagnoses are in Table S1. CA diagnosis was verified by interview of family members. Postmortem mPFC (Brodmann area 46; BA46) from 30 CA individuals (mean age = 36 ± 8.0; 83.3% males; 15 European‐American (EA), 15 African‐American (AA)) who died of cocaine intoxication or cocaine‐related cardiovascular toxicity and 30 age, gender, and ethnicity matched controls (mean age = 35 ± 7.5; 83.3% males; 18 EA, 12 AA), who died of heart disease, other natural cause or non‐CNS trauma, were obtained from the University of Miami Miller School of Medicine Brain Endowment Bank^TM^ (RRID:SCR_00872). Specimens of cerebellum from some CA subjects were also obtained.

### 2.2 Blood samples from CA individuals

De‐identified genomic DNA (gDNA) from EBV‐transformed lymphoblastoid cell lines of EA (*n *= 84; 50.3% male) and AA (*n *= 84; 52.1% male) subjects who met DSM‐IV criteria for CA were acquired from the Rutgers University Cell and DNA Repository (RUCDR, RRID:SCR_010624) (Infinite Biologics, Piscataway, NJ, USA) through the NIDA Center for Genetic Studies in conjunction with Washington University in Saint Louis and the RUCDR. gDNA was diluted to 20 ng/μl in sterile water before use in genotyping experiments.

### 2.3 Neuronal nuclei isolation

Observations in human postmortem tissues suggest L1 retrotransposition events occur more often in neurons than in glia (Coufal et al., 2009; Upton et al., 2015). Therefore, a modified method of Jiang, Matevossian, Huang, Straubhaar, and Akbarian (2008) was used to isolate neuronal (NeuN‐positive) and non‐neuronal (NeuN‐negative) nuclei from frozen mPFC tissue. Six pools of frozen postmortem mPFC (~10–15 mg wet weight/individual), each pool containing mPFC of either 10 cocaine overdose victims (3 pools) or 10 controls (3 pools), were thawed and lysed simultaneously by homogenization in ice‐cold 0.32M sucrose solution containing Triton X‐100, lysates layered onto a 1.8M sucrose cushion and nuclei pelleted by ultracentrifugation (~107,000 *g*, 2.5 hr, 4°C). Pelleted nuclei were suspended in 1× phosphate‐buffered saline containing 3 mM MgCl_2_, labeled with an AlexaFluor‐488‐conjugated anti‐NeuN antibody (Millipore Cat. No: MAB377×, RRID:AB_2149209, Temecula, CA, USA), counterstained with diamidino‐2‐phenylindole (Thermo‐Fisher Scientific, Waltham, MA, USA) and strained through a 30 μm filter‐cap tube (Becton‐Dickinson, Franklin Lakes, NJ, USA) as described (Jiang et al., 2008). Labeled nuclei were sorted into NeuN‐positive and NeuN‐negative populations on an AriaII fluorescence‐activated cell sorter (Beckman‐Coulter, Brea, CA, USA). Sorted nuclei were then pelleted by centrifugation (2000 *g*, 30 min, 4°C), lysed in 1× proteinase K digestion buffer (50 mmol/L Tris, pH 8.0, 100 mmol/L EDTA, 100 mmol/L NaCl, 1% SDS, 0.857 μg/μl proteinase K), incubated at 56°C for 16 hr, and gDNA purified by chloroform extraction and ethanol precipitation in the presence of 20 μg molecular biology grade glycogen (Roche, Indianapolis, IN, USA).

### 2.4 L1 amplification and sequencing

Purified gDNA from NeuN‐positive nuclei was subjected to L1‐seq essentially as described (Ewing & Kazazian, 2010), except primary PCR was done using 25 ng of gDNA template for each of the eight hemi‐specific degenerate primer reactions. We then performed the secondary PCR to add sequencing adapters for next generation sequencing (NGS) (Ewing & Kazazian, 2010). Amplicon libraries were sequenced using 100 nucleotide (nt) single‐end reads in one lane (per pool library) of an Illumina HiSeq 2000.

### 2.5 L1‐seq bioinformatics

Two analyses were done. The first analysis used the “original” version of the published L1‐seq bioinformatics program pipeline (Ewing & Kazazian, 2010). We used bowtie2‐2.1.0 (Langmead & Salzberg, 2012) to trim 10 and 26 nts from the 5' and 3' ends, respectively, of each 100 nt read. Resultant 64 nt reads with Q‐value ≥ 30 at each nucleotide position were aligned to the hg19 reference genome build. Samtools‐0.1.19 (RRID:SCR_002105; Li et al., 2009) converted aligned reads to BAM files. The “original” L1‐seq bioinformatics program (Ewing & Kazazian, 2010), adjusted for a 64 nt read window, then determined the locations of known and previously uncataloged (novel) L1 insertions. Novel L1 insertions were defined as read “peaks” that did not align to known reference (KR) or known non‐reference (KNR) L1s. Quality control metrics were that the L1 was novel, the reads aligned well to the reference genome (“mapq” ≥ 30 and “mapscore” > 0.5) and at least one pooled population had greater than 5 reads, of which at least two were unique (“maxcount” > 5, “maxuniq” > 1). Novel L1s were then compared for overlaps and differences among the pools of sequenced NeuN‐positive libraries.

The second L1‐seq bioinformatics analysis used a new version of the L1‐seq bioinformatics program pipeline (unpublished, available at https://github.com/adamewing/l1seq). Previously uncataloged (novel) L1 insertion sites identified by this L1‐seq pipeline were filtered for high quality using Excel (Microsoft, Redmond, WA, USA). Quality control metrics for a putative, novel L1 insertion were that the reads aligned well to the reference genome (“mean mapq”≥30, “mappability”>0.5), had greater than 98% average percent match (“mean matchpct”>0.98), at least six total reads across pooled populations (“total reads”>5) and at least two unique reads in a “peak” (“unique alignments”>1). Because the new L1‐seq analysis was done at a later time, the annotations of KNR L1s in the databases were more current. This resulted in some of the “novel” L1s detected using the “original” L1‐seq moving into the KNR L1 category.

### 2.6 LINE‐1 validations

We used Primer3 (RRID:SCR_003139; Rozen & Skaletsky, 2000) to design genome‐specific “filled site” (FS, containing an L1) and “empty site” (ES) primers for confirmatory PCR (Ewing & Kazazian, 2010). We attempted to validate novel L1 insertions that were “*intra*genic”; defined as an L1 located within an intron, an exon, or within 500 bp of the transcription start site or 3'UTR (Ewing & Kazazian, 2010). We initially used a random number generator within an Excel spreadsheet to select row numbers of L1s identified by the “original” L1‐seq bioinformatics analysis for PCR validation. We attempted to validate novel L1 insertions detected by the “original” L1‐seq analysis that were *intra*genic because these are most likely to disrupt gene function. Appendix S2 shows the list of all L1 validations attempted (successful highlighted in green).

We prepared libraries for L1‐seq using gDNA purified directly from neuronal and non‐neuronal nuclei. Due to a paucity of gDNA remaining after L1‐seq library constructions, we whole‐genome amplified (WGA) each sample's remaining gDNA before attempting PCR validations. The gDNA input into L1‐seq validation reactions was amplified by multiple displacement amplification performed on 10 ng of each NeuN‐positive or NeuN‐negative gDNA sample using a Repli‐G mini kit (Qiagen, Hilden, Germany). PCR reactions were 25 μl containing 1X GoTaq Hot Start Master Mix (Promega, Madison, WI, USA), 10 ng WGA gDNA, and 0.2 μmol/L primers. Cycling parameters were 95°C–2 min 30 s, 35 cycles of 95°C–30 s, 50°C–30 s, 72°C–2 min, then 72°C–5 min, 4°C soak. We used two initial primer pairings: FS with ES, and FS with L1HsTAILSP1AP2 (L1HsT, 5′‐GGG‐AGA‐TAT‐ACC‐TAA‐TGC‐TAG‐ATG‐ACA‐C‐3'), which would amplify the genomic region surrounding or including, respectively, a putative L1 insertion (Ewing & Kazazian, 2010). In some instances, we performed nested PCR using the parameters above with 2 μl of the initial PCR (FS‐L1HsT primer pairing) as template and nested filled site (FSn) and L1‐specific (L1HsG, 5′‐TGC‐ACA‐TGT‐ACC‐CTA‐AAA‐CTT‐AG‐3′) primers. Amplicons were separated by agarose gel electrophoresis, excised, purified, cloned into pCRII‐TOPO (Thermo‐Fisher Scientific) and ligation products transformed into *E. coli*. Plasmid DNA containing putative L1 insertions were Sanger sequenced using big‐dye chemistry. Resultant sequences were queried against hg19 using BLAT (RRID:SCR_011919; Kent, 2002). To determine the occurrence of confirmed L1 inserts among individuals of a pool and/or regional brain mosaicism of an L1, PCR was performed on bulk gDNA purified from either a second mPFC fragment from each individual and/or from cerebellum of the same individual with the mPFC L1 insert, respectively.

### 2.7 Droplet digital PCR (ddPCR): allele frequency determination

We used L1Hs‐ and gene‐specific primers designed to amplify 200–250 bp products. An L1Hs‐specific probe sequence was as described (White, McCoy, Streva, Fenrich, & Deininger, 2014) except the 5′ FAM‐labeled probe was double‐quenched with 3′ Iowa Black and internal ZEN quenchers (Integrated DNA Technologies). Approximately 100 ng of gDNA was digested with XmnI in ddPCR master mix at 37°C for 1 hr prior to droplet formation using a QX100 droplet generator (Bio‐Rad, Hercules, CA, USA). Droplets were cycled at 95°C–10 min, 40 cycles of 94°C–30 s, 60°C‐1 min, then 98°C‐10 min, 12°C soak. Amplified droplets were read using a QX200 droplet reader (Bio‐Rad) under absolute quantification settings for FAM (L1 allele probe, unknown) or VIC (*RPPH1* allele probe, reference, Thermo‐Fisher Scientific; Cat no. 4403328) fluorescence. QuantaSoft v1.7.4 (Bio‐Rad) was used for data analysis and graph generation.

### 2.8 ddPCR: 3′‐anchored LINE‐1 mRNA detection

Trizol^®^ (Thermo‐Fisher Scientific) extracted total RNA (2 μg) from control or cocaine postmortem mPFC with RNA Integrity Numbers ≥6.5, assessed by Agilent RNA 6000 Nanochips with a Bioanalyzer 2100, were heat denatured with random hexamers (50 ng/reaction) and a (SalNot)oligo(dT)_25_VN primer (0.5 μg/reaction; 5′‐GCT‐AGT‐CGA‐CGC‐GGC‐CGC‐A(T_25_)VN‐3′). Ice‐quenched RNA samples were converted to cDNAs using Superscript III™ (Thermo‐Fisher Scientific) in 20 μl reverse transcription reactions incubated at 25°C–5 min, 42°C–5 min, 50°C–30 min, and then 55°C‐30 min. Reactions were terminated at 70°C–15 min. We diluted the cDNA to 0.5 ng/μl RNA equivalents before ddPCR using 1 ng RNA equivalents as input. We detected L1 mRNA with our double‐quenched, FAM‐labeled L1 probe (White et al., 2014), the L1HsT primer and a SalNot adaptmer primer (5′‐GCT‐AGT‐CGA‐CGC‐GGC‐CGC‐AT‐3′). VIC‐labeled human *GAPDH* (in multiplex), FAM‐labeled human *ACTB* or *TBP* (in simplex) gene expression primer‐probe assay (Thermo‐Fisher Scientific, cat nos. 4326317E, 4333672F or Hs00427620_m1, respectively) were used as reference. Parameters for ddPCR were as described for “Allele Frequency Determination”. Control reactions included cDNA reactions without Superscript III™ reverse transcriptase and no template (water) controls. QuantaSoft v1.7.4 (Bio‐Rad) was used for data analysis.

### 2.9 Database for annotation, visualization and integrated discovery (DAVID) and PANTHER analyses

The L1‐seq bioinformatics analyses generated lists of genomic L1 positions within each studied population. We employed the DAVID (version 6.7; RRID:SCR_001881; Huang, Sherman, & Lempicki, 2009) or PANTHER (version 10.0; RRID:SCR_004869; Mi, Poudel, Muruganujan, Casagrande, & Thomas, 2016) algorithms to analyze lists of genes with KNR or putatively novel L1 insertions for enrichment of gene ontology (GO) terms or Kyoto Encyclopedia of Genes and Genomes (KEGG) pathways. For PANTHER, only the curated “slim” GO term libraries and PANTHER pathways were queried for statistical over‐representation. Because the L1 annotation databases were most current for the “new L1‐seq” analysis, we present DAVID and PANTHER analyses for gene lists generated by the “new L1‐seq” analysis only.

Gene lists input for each L1‐seq analysis corresponded to genes with KNR or putatively novel L1s found in the control and/or cocaine population (Appendix S3). For each list, genes with L1s that overlapped the two populations were excluded or included in the analyses.

### 2.10 Statistics

DAVID employs a hypergeometric distribution to calculate fold enrichment and *p*‐values (Huang et al., 2009; see Appendix S1 for equations). PANTHER employs a binomial distribution to calculate fold enrichment and *p*‐values (Mi et al., 2016; see Appendix S1 for equations). Each algorithm adjusted the “raw” *p*‐values using the Bonferroni correction for multiple testing.

Graphs and statistical analyses of ddPCR were generated using JMP Pro 12 (SAS, Cary, NC, USA) software. Fisher's exact test was done with GraphPad Quickcalcs software at http://graphpad.com/quickcalcs/contingency2/. All statistical tests were two‐sided.

## 3 Results

### 3.1 L1‐seq bioinformatics analyses

Our rates of L1 detection were consistent with previous estimates for segregating L1Hs elements expected in a population of 60 (Ewing & Kazazian, 2010). Under the stringency metrics used, the “original” and “new” L1‐seq analyses detected 1602 and 1616 total L1s (KNR plus novel) across all samples (both CA and control populations; Appendix S4). Moreover, we detected totals of 1117 (“original” analysis) and 853 (“new” analysis) novel L1s across all samples; corresponding to ~19 and ~14 previously uncataloged L1s per sequenced individual, on average, respectively. Notably, 427 of the novel L1s were detected by both L1‐seq analyses (Appendix S4). For the “original” L1‐seq analysis, more *intra*genic novel L1s were found only in the cocaine population (118 vs. 98), but more *inter*genic novel L1s were found only in the control population (130 vs. 101) such that the total number of novel L1s between the two populations did not differ significantly (Fig. S1). For the “new” L1‐seq analysis, more total novel L1s were found only in the cocaine population (90 *intra*genic and 115 *inter*genic; 205 total) than were found only in the control population (82 *intra*genic and 108 *inter*genic; 190 total) (Fig. S1), however, the difference was not statistically significant (Fisher's exact test, *p *=* *.919).

### 3.2 PCR confirmations

We attempted confirmations on randomly selected, previously uncataloged *intra*genic L1s detected by the “original” version of the L1‐seq bioinformatics pipeline (Ewing & Kazazian, 2010). We initially obtained low rates of PCR confirmation (~19%; 12/64; Appendix S2). This rate included failed attempts at validation of putatively novel L1 insertions using a low stringency of two unique reads and mapscore≥0.5. After we adjusted our stringency metrics (stated above), the effective rate of L1 confirmations increased to 39% (11/28) suggesting that many of the L1s we first attempted to validate were false positives or true low frequency *de novo* somatic L1 mutations. Although some of the detected L1s may be false positives, ultimately, our relatively low rates of confirmation may be, in part, due to allelic dropout after whole‐genome amplification of the gDNA before validation PCR.

**Figure 1:**
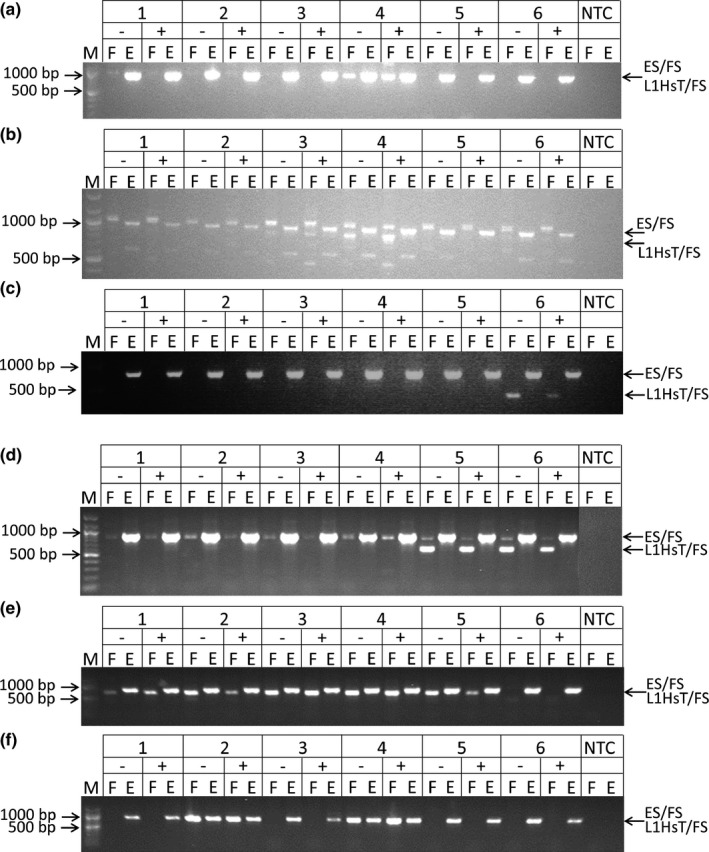
Confirmation of novel L1 RTP insertions in various genes. Gel images showing the “filled site” (“F”; L1HsT/FS primer pair) and “empty site” (“E”; ES/FS primer pair) PCR amplicons. The L1 insertions were detected in both the NeuN‐negative (“−”) and NeuN‐positive (“+”) gDNA reactions. (a) Williams‐Beuren Syndrome chromosome region 17 (*WBSCR17*). (b) Ten‐eleven translocation (TET) methylcytosine dioxygenase 2 (TET2). (c) Syntabulin (SYBU). (d) Disabled (Dab), reelin signal transducer, homolog 1 (DAB1). (e) Kelch like‐1 (KLHL1). (f) TBC1 domain containing kinase (TBCK). The KLHL1 L1 was found in all pools except pool 6, although it was not initially detected in pools 1, 2 and 6. In contrast to KLHL1, L1s in WBSCR17, TET2, SYBU, DAB1 and TBCK were confirmed in the pools in which each predicted L1 insert was initially detected. Lane “M” is the NEB 100 bp marker. Lanes 1, 2, and 5 are control populations, lanes 3, 4, and 6 are CA populations. Minus or plus signs indicate amplicons of gDNA from NeuN‐negative (not sequenced, non‐neuronal) or NeuN‐positive (sequenced, neuronal) nuclei, respectively

L1 retrotransposons were confirmed in Williams‐Beuren Syndrome chromosome region 17 (*WBSCR17,* Figure 1a), ten‐eleven translocation methylcytosine dioxygenase 2 (*TET2,* Figure 1b), syntabulin (*SYBU,* Figure 1c), disabled reelin signal transducer, homolog 1 (*DAB1*, Figure 1d), kelch like‐1 (*KLHL1*, Figure 1e), and TBC1 domain containing kinase (*TBCK*, Figure 1f). L1s were also confirmed in ataxin 1 (*ATXN1)*, CCCTC‐binding factor *(CTCF)*, dachshund family transcription factor 2 (*DACH2)*, DEXD/H‐box helicase 58 (*DDX58)*, macro domain containing 2 (*MACROD2)*, and parathyroid hormone 2 receptor (*PTHR2)* (data not shown). All of these L1 insertions occur within an intron or promoter region of various gene transcripts (data not shown). Those near intron‐exon junctions (*WBSCR17*,* TBCK*) did not interrupt the branch point or splice site (data not shown). Some overlapped DNAseI hypersensitivity sites (*TET2*,* SYBU*), were near binding sites for transcription factors (*TET2*,* SYBU, KLHL1, ATXN1, CTCF, MACROD2*) or were near a histone 3 lysine 27 acetylation peak (*SYBU, ATXN1*; data not shown).

The L1‐seq pipeline annotates L1s as novel if they are not found in the reference genome and are not found in any of the L1 databases (dbRIP, EUL1db, etc.) or various L1 publications. Studies of L1s in the whole genome sequencing data of phase 1 of the 1000 genomes project have been published (Ewing & Kazazian, 2011; Stewart et al., 2011), and the L1s detected in these publications were annotated as known non‐reference L1s at the time of our L1‐seq analyses. Subsequently, in response to reviewer comments, we cross‐referenced our list of detected L1s with the 2015 publication on structural variants in the 1000 genomes project (Sudmant et al., 2015). Although the *SYBU*,* DAB1, KLHL1, TBCK, PTHR2,* and *MACROD2* L1s were found in the 1000 genomes project, the *TET2, WBSCR17, ATXN1, CTCF, DDX58,* and *DACH2* L1s confirmed in our study were not among those in the phase 3 data of the 1000 genomes project. Therefore, as far as can be determined at this point, the latter L1s could be private mutations.

We attempted to amplify the 5′ end of some of the identified L1′s in our study without success. The approach utilized for each insertion involved use of the reverse‐complement of the L1Hs‐specific primer paired with the empty site genome‐specific primer in a nested PCR to amplify the 5′ end. This approach was taken because we amplified the 3′ ends of the L1s for L1‐seq analysis and PCR verifications and do not know how much of the 5′ end is actually present in the detected L1. Thus, whereas we do have the 3′ end and poly‐A tail of the verified L1s, we have not yet confirmed the 5′ end with the target site duplication (if present). The Sanger sequencing results of the cloned L1 3′ ends are in Appendix S5.

The *DAB1* and *TBCK* L1s were found in two individuals, one in the control population and one in the CA population (data not shown). The *WBSCR17, TET2*,* and SYBU* L1 insertions were found in one individual in the CA population (data not shown). There was no evidence for regional mosaicism of the *WBSCR17*,* TET2*, or *SYBU* L1 insertions in the brains of the respective individuals as each L1 insertion detected in mPFC gDNA was also detected in cerebellar gDNA (data not shown). One caveat to these findings is that single cell neuronal gDNA, which may have revealed cellular somatic mosaicism (Evrony et al., 2012), was not assessed.

### 3.3 DAVID analyses

DAVID analyses of gene lists from the control and/or CA populations (Appendix S3) resulted in statistically significant GO term enrichments for L1 insertions into genes in the CA population that were not observed in the control population (Table 1). The GO term “synapse” was nominally significant only when the KNR L1 gene list from the cocaine population was input (Table 1A). Several nucleoside binding (i.e., ATP‐binding) GO terms were enriched when genes harboring KNR L1s from only the control population were input (Table 1A). Genes harboring putative novel L1s specific to the cocaine population identified statistically significant GO term enrichments related to the plasma membrane (Table 1B). None of the other significant GO terms showed population‐specific enrichments. None of the lists identified statistically significant KEGG pathways.

**Table 1:** Results of DAVID analyses

	Gene counts	Percent of input list	Fold enrichments over background	Bonferroni corrected *p*‐values
(A) KNR L1s Gene List – Overlapping genes included	Cocaine (Control)			
GO_cellular component				
GO:0043005~neuron projection	18 (17)	8.29 (8.54)	4.61 (5.00)	8.51E‐05 (5.82E‐05)
GO:0030054~cell junction	19 (16)	8.76 (8.04)	3.21 (3.11)	5.93E‐03 (4.11E‐02)
GO:0042995~cell projection	21 (NA)	9.68 (NA)	2.64 (NA)	2.99E‐02 (NA)
GO:0045202~synapse	14 (NA)	6.45 (NA)	3.45 (NA)	5.09E‐02 (NA)
GO_molecular function				
GO:0032559~adenyl ribonucleotide binding	(NA) 34	(NA) 17.09	(NA) 2.05	(NA) 2.44E‐02
GO:0030554~adenyl nucleotide binding	(NA) 35	(NA) 17.59	(NA) 2.00	(NA) 2.86E‐02
GO:0001883~purine nucleoside binding	(NA) 35	(NA) 17.59	(NA) 1.97	(NA) 3.84E‐02
GO:0001882~nucleoside binding	(NA) 35	(NA) 17.59	(NA) 1.96	(NA) 4.38E‐02
(B) Novel L1s gene list – overlapping genes included				
GO_cellular component				
GO:0030054~cell junction	25 (24)	8.96 (8.86)	3.05 (2.99)	5.97E‐04 (1.54E‐03)
GO:0044459~plasma membrane part	58 (NA)	20.79 (NA)	1.67 (NA)	1.59E‐02 (NA)
GO:0005886~plasma membrane	86 (NA)	30.82 (NA)	1.44 (NA)	2.12E‐02 (NA)
(C) Novel L1s gene list – overlapping genes excluded				
GO_cellular component				
GO:0042995~cell projection	(NA) 12	(NA) 16.44	(NA) 4.15	(NA) 1.77E‐02

NA, Not applicable.

### 3.4 PANTHER analyses: Slim GO term libraries and PANTHER pathways

PANTHER analyses of gene lists (Appendix S3) revealed additional statistically significant GO terms and pathways among genes in the CA population that were not significant in the control population (Table 2). Notably, the PDGF signaling pathway was significant when KNR L1 gene list from the cocaine, but not the control, populations were input (with or without the genes that overlapped both populations; Table 2A and 2B). Similarly, the GO term “signal transduction” was significant when the novel L1 gene list form the cocaine, but not control, population was input (but only when overlapping genes were excluded; Table 2C). Other interesting cocaine population‐specific GO terms were “hydrolase activity, acting on ester bonds” (Table 2B), “enzyme regulator activity” (Table 2C) and “transmembrane transporter activity” (Table 2D). The PANTHER pathway “Endothelin signaling pathway” was also cocaine population‐specific (Table 2C). Finally, similar to DAVID analysis, the GO term “plasma membrane” was significant when the cocaine population gene list was input into PANTHER (Table 2D).

**Table 2:** Results of PANTHER analyses (GO_slim and pathways)

	Observed gene counts	Expected gene counts	Fold enrichments over background	Bonferroni corrected *p*‐values
(A) KNR L1s Gene List – Overlapping genes excluded	Cocaine (Control)			
PANTHER Pathways				
PDGF signaling pathway (P00047)	5 (NA)	0.36 (NA)	14.08 (NA)	0.00464 (NA)
(B) KNR L1s gene list – overlapping genes included				
GO_biological process				
Heart development (GO:0007507)	NA (8)	NA (1.36)	NA (5.87)	NA (0.0195)
Developmental process (GO:0032502)	NA (37)	NA (18.48)	NA (2)	NA (0.00886)
Intracellular signal transduction (GO:0035556)	26 (23)	10.4 (9.45)	2.5 (2.43)	0.00452 (0.0202)
Signal transduction (GO:0007165)	47 (42)	25.07 (22.79)	1.87 (1.84)	0.00399 (0.0164)
Cell communication (GO:0007154)	53 (48)	28.05 (25.5)	1.89 (1.88)	0.000772 (0.00242)
GO_molecular function				
Hydrolase activity, acting on ester bonds (GO:0016788)	17 (NA)	6.29 (NA)	2.7 (NA)	0.0407 (NA)
PANTHER pathways				
Alpha adrenergic receptor signaling pathway (P00002)	4 (NA)	0.26 (NA)	15.25 (NA)	0.0247 (NA)
PDGF signaling pathway (P00047)	9 (NA)	1.56 (NA)	5.76 (NA)	0.00536 (NA)
(C) Novel L1s gene list – overlapping genes excluded				
GO_biological process				
Signal transduction (GO:0007165)	22 (NA)	9.34 (NA)	2.35 (NA)	0.0227 (NA)
Cell communication (GO:0007154)	25 (NA)	10.46 (NA)	2.39 (NA)	0.00484 (NA)
Cellular process (GO:0009987)	(NA) 46	(NA) 28.54	(NA) 1.61	(NA) 0.00742
GO_molecular function				
Enzyme regulator activity (GO:0030234)	11 (NA)	2.47 (NA)	4.45 (NA)	0.00634 (NA)
Structural constituent of cytoskeleton (GO:0005200)	(NA) 10	(NA) 2.07	(NA) 4.84	(NA) 0.00707
GO_cellular component				
Intracellular (GO:0005622)	(NA) 30	(NA) 13.99	(NA) 2.14	(NA) 0.000786
Cell part (GO:0044464)	(NA) 30	(NA) 14.32	(NA) 2.09	(NA) 0.00126
PANTHER pathways				
Endothelin signaling pathway (P00019)	5 (NA)	0.34 (NA)	14.87 (NA)	0.00384 (NA)
(D) Novel L1s gene list – overlapping genes included				
GO_biological process				
Cyclic nucleotide metabolic process (GO:0009187)	7 (NA)	1.08 (NA)	6.48 (NA)	0.0279 (NA)
Visual perception (GO:0007601)	NA (11)	NA (2.87)	NA (3.83)	NA (0.0401)
Organelle organization (GO:0006996)	NA (20)	NA (7.49)	NA (2.67)	NA (0.018)
Cellular component organization (GO:0016043)	NA (35)	NA (15.82)	NA (2.21)	NA (0.00231)
Cellular component organization or biogenesis (GO:0071840)	NA (36)	NA (17.26)	NA (2.09)	NA (0.00597)
Transport (GO:0006810)	NA (54)	NA (32.44)	NA (1.66)	NA (0.0268)
Localization (GO:0051179)	NA (56)	NA (34.19)	NA (1.64)	NA (0.03)
Cellular process (GO:0009987)	NA (121)	NA (87.98)	NA (1.38)	NA (0.00436)
GO_cellular component				
Integral to membrane (GO:0016021)	11 (NA)	3.33 (NA)	3.3 (NA)	0.0296 (NA)
Plasma membrane (GO:0005886)	16 (NA)	6.43 (NA)	2.49 (NA)	0.0414 (NA)
Cell part (GO:0044464)	NA (60)	NA (40.53)	NA (1.48)	NA (0.0496)
GO_molecular function				
Transmembrane transporter activity (GO:0022857)	28 (NA)	13.64 (NA)	2.05 (NA)	0.0491 (NA)

NA, Not applicable.

### 3.5 Allele frequency determination by ddPCR

**Figure 2:**
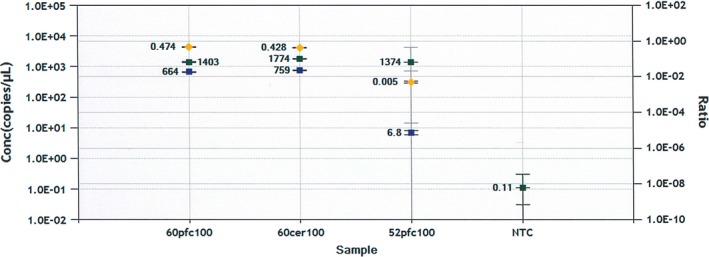
ddPCR allele frequency of the L1 in *SYBU*. Graph showing the absolute copy numbers of *SYBU*‐L1 (blue squares) and *RPPH1* (green squares) genes, as well as the ratios (*SYBU*‐L1:RPPH1, orange diamonds), detected in 100 ng of XmnI‐digested gDNA from mPFC (“60pfc100″) and cerebellum (“60cer100″) of CA individual 60, who had the *SYBU*‐L1, or from 100 ng of XmnI‐digested gDNA from mPFC (“52pfc100”) of CA individual 52, who did not have the *SYBU*‐L1. NTC is the no template control

In theory, if a novel L1 insertion created cellular somatic mosaicism in the mPFC, the ratio of the unknown (L1) to the reference (*RPPH1*) allele assessed by ddPCR would be less than 50% (the expected value for an L1 that is heterozygous in the diploid genome of every cell of the examined tissue). Both the novel *SYBU* L1 (Figure 2) and the KNR L1 in *JAK2* (Iskow et al., 2010; used as a positive control, Fig. S2) had unknown/reference ratios of about 50% suggesting each individual is heterozygous for these L1s. The *SYBU* L1 was not found in blood gDNA of 84 EA or 84 AA cocaine addicts assessed by confirmatory PCR (data not shown). This contrasts with the known nonreference L1 in *JAK2* (Iskow et al., 2010) being found in blood gDNA from 3 of 84 EAs with CA (data not shown). Thus, current data suggest that either the *SYBU* L1 arose early during neurogenesis or both L1s (*SYBU* and *JAK2*) are polymorphic in the germline.

### 3.6 L1 mRNA levels determination by ddPCR

**Figure 3:**
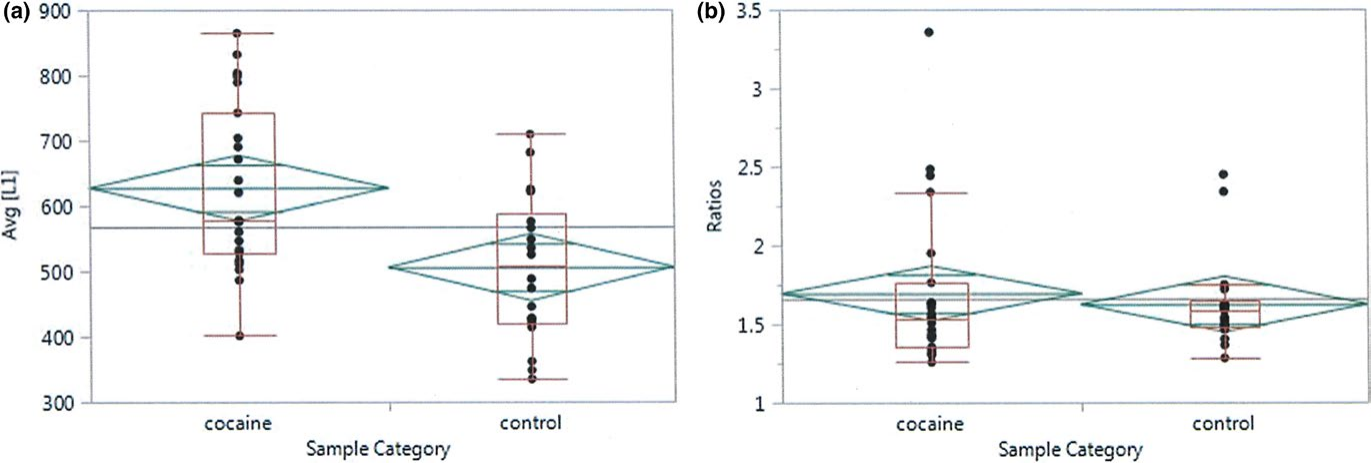
L1 mRNA detection in mPFC of control and CA individuals by ddPCR. Representative graphs showing box plots (red) and mean diamonds (green) for determinations of (a) absolute values of L1 mRNA (each point is the average of three independent determinations); Wilcoxon Rank Sum, *p* = .0064, (b) ratios of average L1 mRNA levels (shown in (a)) to the geometric means (GEO mean) of three reference normalizers (*GAPDH, ACTB* and *TBP*); Wilcoxon Rank Sum, *p *= .547

Three independent ddPCR experiments indicated significantly higher absolute levels of L1 mRNA transcripts in CA mPFC than control mPFC (Figure 3a; Wilcoxon Rank Sums, *p*‐values = 0.0062, 0.0076, and 0.0084 for each respective analysis). However, when absolute L1 mRNA levels were corrected for input cDNA quality and quantity by normalization with *GAPDH, ACTB*,* TBP* or the geometric mean (GEO mean) of the three different normalizers, relative ratios (L1/*normalizer*) were not significantly different between the two populations (Wilcoxon Rank Sums, *p*‐value = 0.271 (*GAPDH*), 0.149 (*ACTB*), 0.0785 (*TBP*), and 0.547 (GEO mean; Figure 3b)).

## 4 Discussion

The confirmed novel L1 insertions in *WBSCR17*,* TET2*, and *SYBU* were in both the NeuN‐negative (non‐neuronal) and NeuN‐positive (neuronal) gDNA of a single CA pool and/or individual (Figure 1a–c; data not shown) suggesting they are polymorphic in the germline or occurred somatically *de novo* during the early neurodevelopment of the individual(s). The *KLHL1*,* DAB1*, and *TBCK* L1 insertions were observed in multiple populations and/or individuals (Figure 1d–f; data not shown) suggesting they are polymorphic in the germline. Our ddPCR data indicating a *SYBU* L1 frequency of about 50% in both the mPFC and cerebellum (Figure 2) is consistent with PCR data documenting its presence in both the NeuN‐negative and NeuN‐positive gDNA fractions (Figure 1c). Thus, 100% of neurons and glia, heterozygous for this novel L1, are likely affected. The *SYBU* L1 was not detected in gDNA samples from the blood of 84 individuals of European or African descent (data not shown), but was subsequently found in the phase 3 dataset of structural variants of the 1000 genomes project (Sudmant et al., 2015) at very low minor allele frequencies (≤1%) in 2 African (GDW, MSL) and 1 European (IBS) population(s). In contrast, the *TET2* and *WBSCR17* L1s may be private mutations because they were found in one individual and were not found among the L1s in the phase 3 data set of the 1000 genomes project (Sudmant et al., 2015).These data, suggesting rare germline L1 variants (*SYBU*) or potentially private L1s (*TET2* and *WBSCR17*), are consistent with the hypothesis that polymorphic germline or early developmental *de novo* somatic L1s might be risk factors predisposing an individual to developing CA.

Notably, DNA markers near/within syntabulin (*SYBU)* showed nominally significant association with disease in a cocaine addiction GWAS (Gelernter et al., 2014) and *Sybu* expression was down‐regulated in orbitofrontal cortex of rats after cocaine administration (Winstanley et al., 2007). Syntabulin is a linker molecule between syntaxin 1A (SYN1A) and the kinesin 1 family member 5B motor protein that delivers SYN1A‐containing vesicles to the presynaptic nerve terminal (Su, Cai, Gerwin, Smith, & Sheng, 2004). SYN1A interaction with the NH_2_‐terminus of the dopamine transporter (DAT), to which cocaine binds, regulates the transporter by suppressing DAT channel activity (Carvelli, Blakely, & DeFelice, 2008). If the observed L1 insertion disrupts SYBU expression, then the potential decrease of SYN1A at nerve terminals could lead to disruption or loss of the SYN1A::DAT interaction promoting dopamine uptake by DAT (Carvelli et al., 2008). An individual with L1 disruption of *SYBU* might have less dopamine in synaptic clefts at baseline leading to a greater relative increase in dopamine after an initial, acute cocaine self‐administration. This scenario may be consistent with greater reward from cocaine.

An L1 insertion was also confirmed in *TET2* (Figure 1b), which encodes a protein that converts 5‐methylcytosine (5‐mC) to 5‐hydroxymethylcytosine (5‐hmC); this can be the first step in demethylation of DNA (Dahl, Grønbæk, & Guldberg, 2011) and contribute to the dynamic state of DNA methylation. TET2 activity may be especially important in the brain where 5‐hmC modified DNA is abundant (Dahl et al., 2011). In mice, conditioned place preference (CPP) for cocaine elicited hypomethylation of DNA in the PFC, but not in the nucleus accumbens (NAcc) (Tian et al., 2012). Although the effects of cocaine on TET2 activity have not been tested directly, the conversion of 5‐mC to 5‐hmC by TET2 in the cerebral cortex may play a part in changing the DNA methylation state in response to cocaine. If so, weaker reward to an initial cocaine self‐administration might be evoked in an individual with an L1‐disrupted *TET2* gene because the balance toward DNA hypomethylation in the PFC might not be firmly established.

The L1s in *DAB1* (Figure 1d) and *TBCK* (Figure 1f) are intriguing because the encoded proteins interact with signal transduction pathways that are affected by cocaine (Sutton & Caron, 2015; Teixeira et al., 2014). *Dab1* knockout mice exhibit greater locomotor sensitization in response to repeated cocaine administration than their wild type counterparts (Teixeira et al., 2014). *TBCK* regulates the mammalian target of rapamycin pathway (Liu, Yan, & Zhou, 2013) that is documented as being important for cocaine (i.e., dopamine)‐mediated signal transduction (Shi et al., 2014; Sutton & Caron, 2015). Importantly, both of these L1s are KNR L1s that are polymorphic in the germline, meaning that they can be inherited. Thus, if functionally disruptive of the gene(s), an individual who inherits these L1s might be more prone to developing CA than someone who does not.

Several signal transduction‐related GO terms and pathways were identified by PANTHER as being statistically over‐represented in the cocaine population (Table 2). The PDGF signaling pathway was over‐represented in the cocaine population, both with and without the exclusion of those KNR L1s that overlapped the populations (Table 2A and 2B). One of these KNR L1s within the PDGF signaling pathway is found in *JAK2*, the same L1 that was observed in 3 of 84 EA individuals with CA (data not shown). Interestingly, cocaine signaling through the σ‐1R receptor induced PDGF in the brain 24 hours after cocaine administration and induction of PDGF led to increased vascular permeability of the blood‐brain barrier (Yao, Duan, & Buch, 2011). The endothelin signaling pathway was also identified by PANTHER when cocaine‐specific genes harboring putatively novel L1s was used as input (Table 2C). An acute effect of cocaine is the release of endothelin‐1 (ET‐1), a potent vasoconstrictor, with elevated levels observed within 6 hours and normal levels returning by 24 hours (Pradhan, Mondal, Chandra, Ali, & Agrawal, 2008). Activation of the ET_A_R of ET‐1 decreases NO, a vasodilator, production by suppressing eNOS expression. Thus, acute release of ET‐1 would cause a severe vasoconstriction of brain vasculature whereas delayed release of PDGF would cause relaxation and hyper‐permeability of the vasculature. When intact, these two pathways could cause vasospasm in the brains of cocaine users, possibly resulting in stroke (Treadwell & Robinson, 2007). That both PDGF and ET‐1 signaling pathways were identified by PANTHER suggests that some individuals in our cocaine cohort might have gene disruptions by L1s within these pathways which would likely be neuroprotective after cocaine use.

*Ex vivo* mouse brain (Maze et al., 2011) and neuronal cell line (Okudaira et al., 2014) experiments suggested increased L1 transcription and retrotransposition, respectively, occurs after cocaine exposure. Our data indicated no significant increase in relative levels of L1 mRNA in mPFC of CA individuals as compared to controls (Figure 3). Thus, with the caveat that we could not control the dosing or timing of an individual's cocaine exposure before death, these data are inconsistent with previous studies in mice showing elevated L1 transcripts after repeated cocaine exposures (Maze et al., 2011).

Attempts to confirm several putatively novel L1 insertions were unsuccessful possibly due to characteristics of the L1 retrotransposition process, including L1‐mediated deletion of flanking gDNA and 3′ transductions, which occur frequently (Richardson et al., 2015; Tubio et al., 2014). The use of gDNA produced via WGA may have caused chimeras and/or rearrangements of the source material (Evrony et al., 2012), making PCR validation difficult. Semi‐nested PCR can detect 1 in 1000 cells that have an L1 integration event (Ewing et al., 2015). In the context of this study, L1s with low NGS read counts, if real, are possibly *de novo* somatic mutations, perhaps being present in a small fraction of neurons (Evrony et al., 2012). Although somatic events may have been detected by NGS of the original non‐WGA source gDNA, the amount of gDNA containing such a somatic insertion for validation may be negligible, even after WGA.

Limitations to our study include i) small sample size; correctable in subsequent studies. With a medium effect size, a population of 60 cocaine addicted persons and 60 control individuals gives 80% power to detect an L1 with a minor allele frequency of 5%. It is likely that L1s with low minor allele frequencies in each population were undetected because our population sizes were 30 individuals per group and we pooled individuals. ii) An unknown number of neurons carrying any *de novo* L1; addressable by iterative single mPFC neuron sequencing of each L1 locus. iii) Unknown impact of any L1 on neuronal function; addressable by examining cultured neurons (made heterozygous for the L1 insertion) at baseline and after incubation with cocaine. Blockade of gliotransmission in mice prevents cocaine reinstatement (Turner, Ecke, Briand, Haydon, & Blendy, 2013). Thus, we cannot exclude the potential importance of inherited L1‐mediated gene disruptions on glial cell function and relapse to cocaine taking. Additionally, polysubstance abuse is consistent with epidemiologic studies of CA (Regier et al., 1990; Trenz et al., 2012) and with studies of treatment‐seeking CA patients (Ahmadi et al., 2009). Thus, although the CA subjects died of cocaine overdose and had histories of CA, it is highly probable that most were addicted to multiple substances, not only cocaine. This creates some doubt in assigning L1 CA patient‐control differences strictly to cocaine. It is unknown whether these CA findings would extend to other commonly abused drugs; however, the *in vitro* data of Okudaira et al. (2014) indicate that in neuronal cell lines, methamphetamine, but not ethanol or barbiturates, may also induce L1 retrotransposition. Moszczynska, Flack, Qiu, Muotri, and Killinger (2015) also showed that methamphetamine increased L1 expression and retrotransposition. It is highly unlikely that a substantial number of postmortem brain specimens can be obtained from individuals who have had no addiction other than to cocaine, given the addiction co‐morbidities in epidemiological studies. Although most L1 insertions detected in our study are likely polymorphic in the germline or occurred *de novo* at an early developmental stage, we cannot fully exclude the possibility that some small fraction of L1 mutations arose *de novo* somatically as a consequence of cocaine use. An animal model in which cocaine alone is self‐administered may help address these issues.

In summary, although a small fraction of the novel L1s detected by our study may be a molecular consequence of cocaine taking by CA individuals, most are likely germline or developmental *de novo* mutations acting as antecedent risk factors for CA. Our current data do not indicate that acutely toxic cocaine use or chronic CA increased L1 transcription or retrotransposition in human mPFC. The presence of novel L1s in genes with enrichments in ontologies and pathways previously associated with the effects of cocaine suggests that L1 retrotransposition is a genetic mechanism warranting further study regarding its potential for influencing risk for CA.

## Conflicts of Interest

HHK is on the scientific board of Transposagen Biopharmaceuticals, Inc. All other authors have no conflicts of interest to declare.

## References

[brb3678-bib-0001] Ahmadi, J. , Kampman, K. M. , Oslin, D. M. , Pettinati, H. M. , Dackis, C. , & Sparkman, T. (2009). Predictors of treatment outcome in outpatient cocaine and alcohol dependence treatment. The American Journal on Addictions, 18(1), 81–86. 19219669 10.1080/10550490802545174 PMC3777818

[brb3678-bib-0002] Alterman, A. I. , McKay, J. R. , Mulvaney, F. D. , & McLellan, A. T. (1996). Prediction of attrition from day hospital treatment in lower socioeconomic cocaine‐dependent men. Drug and Alcohol Dependence, 40(3), 227–233. 8861401 10.1016/0376-8716(95)01212-5

[brb3678-bib-0003] Baillie, J. K. , Barnett, M. W. , Upton, K. R. , Gerhardt, D. J. , Richmond, T. A. , De Sapio, F. , … Faulkner, G. J. (2011). Somatic retrotransposition alters the genetic landscape of the human brain. Nature, 479(7374), 534–537. 22037309 10.1038/nature10531 PMC3224101

[brb3678-bib-0004] Bundo, M. , Toyoshima, M. , Okada, Y. , Akamatsu, W. , Ueda, J. , Nemoto‐Miyauchi, T. , … Iwamoto, K. (2014). Increased L1 retrotransposition in the neuronal genome in schizophrenia. Neuron, 81(2), 306–313. 24389010 10.1016/j.neuron.2013.10.053

[brb3678-bib-0005] Carroll, K. M. , Fenton, L. R. , Ball, S. A. , Nich, C. , Frankforter, T. L. , Shi, J. , & Rounsaville, B. J. (2004). Efficacy of disulfiram and cognitive behavior therapy in cocaine‐dependent outpatients: A randomized placebo‐controlled trial. Archives of General Psychiatry, 61(3), 264–272. 14993114 10.1001/archpsyc.61.3.264 PMC3675448

[brb3678-bib-0006] Carvelli, L. , Blakely, R. D. , & DeFelice, L. J. (2008). Dopamine transporter/syntaxin 1A interactions regulate transporter channel activity and dopaminergic synaptic transmission. Proceedings of the National Academy of Sciences of the United States of America, 105(37), 14192–14197. 18768815 10.1073/pnas.0802214105 PMC2528871

[brb3678-bib-0007] Coufal, N. G. , Garcia‐Perez, J. L. , Peng, G. E. , Marchetto, M. C. , Muotri, A. R. , Mu, Y. , … Gage, F. H. (2011). Ataxia telangiectasia mutated (ATM) modulates long interspersed element‐1 (L1) retrotransposition in human neural stem cells. Proceedings of the National Academy of Sciences of the United States of America, 108(51), 20382–20387. 22159035 10.1073/pnas.1100273108 PMC3251057

[brb3678-bib-0008] Coufal, N. G. , Garcia‐Perez, J. L. , Peng, G. E. , Yeo, G. W. , Mu, Y. , Lovci, M. T. , … Gage, F. H. (2009). L1 retrotransposition in human neural progenitor cells. Nature, 460(7259), 1127–1131. 19657334 10.1038/nature08248 PMC2909034

[brb3678-bib-0009] Dahl, C. , Grønbæk, K. , & Guldberg, P. (2011). Advances in DNA methylation: 5‐hydroxymethylcytosine revisited. Clinica Chimica Acta, 412(11–12), 831–836. 10.1016/j.cca.2011.02.013 21324307

[brb3678-bib-0010] Degenhardt, L. , Singleton, J. , Calabria, B. , McLaren, J. , Kerr, T. , Mehta, S. , … Hall, W. D. (2011). Mortality among cocaine users: A systematic review of cohort studies. Drug and Alcohol Dependence, 113(2–3), 88–95. Review. 20828942 10.1016/j.drugalcdep.2010.07.026

[brb3678-bib-0011] Evrony, G. D. , Cai, X. , Lee, E. , Hills, L. B. , Elhosary, P. C. , Lehmann, H. S. , … Walsh, C. A. (2012). Single‐neuron sequencing analysis of L1 retrotransposition and somatic mutation in the human brain. Cell, 151(3), 483–496. 23101622 10.1016/j.cell.2012.09.035 PMC3567441

[brb3678-bib-0012] Evrony, G. D. , Lee, E. , Mehta, B. K. , Benjamini, Y. , Johnson, R. M. , Cai, X. , … Walsh, C. A. (2015). Cell lineage analysis in human brain using endogenous retroelements. Neuron, 85(1), 49–59. 25569347 10.1016/j.neuron.2014.12.028 PMC4299461

[brb3678-bib-0013] Ewing, A. D. , Gacita, A. , Wood, L. D. , Ma, F. , Xing, D. , Kim, M. S. , … Solyom, S. (2015). Widespread somatic L1 retrotransposition occurs early during gastrointestinal cancer evolution. Genome Research, 25(10), 1536–1545. 26260970 10.1101/gr.196238.115 PMC4579339

[brb3678-bib-0014] Ewing, A. D. , & Kazazian Jr, H. H. (2010). High‐throughput sequencing reveals extensive variation in human‐specific L1 content in individual human genomes. Genome Research, 20(9), 1262–1270. 20488934 10.1101/gr.106419.110 PMC2928504

[brb3678-bib-0015] Ewing, A. D. , & Kazazian Jr, H. H. (2011). Whole‐genome resequencing allows detection of many rare LINE‐1 insertion alleles in humans. Genome Research, 21(6), 985–990. 20980553 10.1101/gr.114777.110 PMC3106331

[brb3678-bib-0016] Feng, Q. , Moran, J. V. , Kazazian Jr, H. H. , & Boeke, J. D. (1996). Human L1 retrotransposon encodes a conserved endonuclease required for retrotransposition. Cell, 87(5), 905–916. 8945517 10.1016/s0092-8674(00)81997-2

[brb3678-bib-0017] Gelernter, J. , Sherva, R. , Koesterer, R. , Almasy, L. , Zhao, H. , Kranzler, H. R. , & Farrer, L. (2014). Genome‐wide association study of cocaine dependence and related traits: FAM53B identified as a risk gene. Molecular Psychiatry, 19(6), 717–723. 23958962 10.1038/mp.2013.99 PMC3865158

[brb3678-bib-0018] Hancks, D. C. , & Kazazian Jr, H. H. (2016). Roles of retrotransposon insertions in human disease. Mobile DNA, 7, 9. Review. 27158268 10.1186/s13100-016-0065-9 PMC4859970

[brb3678-bib-0019] Hazen, J. L. , Faust, G. G. , Rodriguez, A. R. , Ferguson, W. C. , Shumilina, S. , Clark, R. A. , … Baldwin, K. K. (2016). The complete genome sequences, unique mutational spectra, and developmental potency of adult neurons revealed by cloning. Neuron, 89(6), 1223–1236. 26948891 10.1016/j.neuron.2016.02.004 PMC4795965

[brb3678-bib-0020] Huang, W. , Sherman, B. T. , & Lempicki, R. A. (2009). Systematic and integrative analysis of large gene lists using DAVID bioinformatics resources. Nature Protocols, 4(1), 44–57. 19131956 10.1038/nprot.2008.211

[brb3678-bib-0021] Iskow, R. C. , McCabe, M. T. , Mills, R. E. , Torene, S. , Pittard, W. S. , Neuwald, A. F. , … Devine, S. E. (2010). Natural mutagenesis of human genomes by endogenous retrotransposons. Cell, 141(7), 1253–1261. 20603005 10.1016/j.cell.2010.05.020 PMC2943760

[brb3678-bib-0022] Jiang, Y. , Matevossian, A. , Huang, H. S. , Straubhaar, J. , & Akbarian, S. (2008). Isolation of neuronal chromatin from brain tissue. BMC Neuroscience, 9, 42. 18442397 10.1186/1471-2202-9-42 PMC2377267

[brb3678-bib-0023] Kalivas, P. W. (2009). The glutamate homeostasis hypothesis of addiction. Nature Reviews Neuroscience, 10(8), 561–572. 19571793 10.1038/nrn2515

[brb3678-bib-0024] Kampman, K. M. , Alterman, A. I. , Volpicelli, J. R. , Maany, I. , Muller, E. S. , Luce, D. D. , … O'Brien, C. P. (2001). Cocaine withdrawal symptoms and initial urine toxicology results predict treatment attrition in outpatient cocaine dependence treatment. Psychology of Addictive Behaviors, 15(1), 52–59. 11255939 10.1037/0893-164x.15.1.52

[brb3678-bib-0025] Kampman, K. M. , Volpicelli, J. R. , Mulvaney, F. , Rukstalis, M. , Alterman, A. I. , Pettinati, H. , … O'Brien, C. P. (2002). Cocaine withdrawal severity and urine toxicology results from treatment entry predict outcome in medication trials for cocaine dependence. Addictive Behaviors, 27(2), 251–260. 11817766 10.1016/s0306-4603(01)00171-x

[brb3678-bib-0026] Kano, H. , Godoy, I. , Courtney, C. , Vetter, M. R. , Gerton, G. L. , Ostertag, E. M. , & Kazazian Jr, H. H. (2009). L1 retrotransposition occurs mainly in embryogenesis and creates somatic mosaicism. Genes & Development, 23(11), 1303–1312. 19487571 10.1101/gad.1803909 PMC2701581

[brb3678-bib-0027] Kazazian Jr, H. H. , Wong, C. , Youssoufian, H. , Scott, A. F. , Phillips, D. G. , & Antonarakis, S. E. (1988). Haemophilia A resulting from de novo insertion of L1 sequences represents a novel mechanism for mutation in man. Nature, 332(6160), 164–166. 2831458 10.1038/332164a0

[brb3678-bib-0028] Kent, W. J. (2002). BLAT–the BLAST‐like alignment tool. Genome Research, 12(4), 656–664. 11932250 10.1101/gr.229202 PMC187518

[brb3678-bib-0029] Koob, G. F. , & Volkow, N. D. (2010). Neurocircuitry of addiction. Neuropsychopharmacology, 35(1), 217–238. Review. Erratum in: Neuropsychopharmacology. 35(4):1051. 19710631 10.1038/npp.2009.110 PMC2805560

[brb3678-bib-0030] Lander, E. S. , Linton, L. M. , Birren, B. , Nusbaum, C. , Zody, M. C. , Baldwin, J. , … International Human Genome Sequencing Consortium (2001). International Human Genome Sequencing Consortium Initial sequencing and analysis of the human genome. Nature, 409(6822), 860–921. Erratum in: Nature 2001 412(6846):565. Nature 2001 411(6838):720. 11237011 10.1038/35057062

[brb3678-bib-0031] Langmead, B. , & Salzberg, S. L. (2012). Fast gapped‐read alignment with Bowtie 2. Nature Methods, 9(4), 357–359. 22388286 10.1038/nmeth.1923 PMC3322381

[brb3678-bib-0032] Li, H , Handsaker, B , Wysoker, A , Fennell, T , Ruan, J , Homer, N & 1000 Genome Project Data Processing Subgroup (2009). The Sequence Alignment/Map format and SAMtools. Bioinformatics, 25(16), 2078–2079. 19505943 10.1093/bioinformatics/btp352 PMC2723002

[brb3678-bib-0033] Liu, Y. , Yan, X. , & Zhou, T. (2013). TBCK influences cell proliferation, cell size and mTOR signaling pathway. PLoS ONE, 8(8), e71349. eCollection 2013. 23977024 10.1371/journal.pone.0071349 PMC3747267

[brb3678-bib-0034] Macia, A. , Widmann, T. J. , Heras, S. R. , Ayllon, V. , Sanchez, L. , Benkaddour‐Boumzaouad, M. , … Garcia‐ Perez, J. L. (2017). Engineered LINE‐1 retrotransposition in non‐dividing human neurons. Genome Research, 27(3), 335–348. doi: 10.1101/gr.206805.116 27965292 10.1101/gr.206805.116 PMC5340962

[brb3678-bib-0035] Martin, S. L. (2010). Nucleic acid chaperone properties of ORF1p from the non‐LTR retrotransposon, LINE‐1. RNA Biology, 7(6), 706–711. 21045547 10.4161/rna.7.6.13766 PMC3073329

[brb3678-bib-0036] Mathias, S. L. , Scott, A. F. , Kazazian Jr, H. H. , Boeke, J. D. , & Gabriel, A. (1991). Reverse transcriptase encoded by a human transposable element. Science, 254(5039), 1808–1810. 1722352 10.1126/science.1722352

[brb3678-bib-0037] Maze, I. , Feng, J. , Wilkinson, M. B. , Sun, H. , Shen, L. , & Nestler, E. J. (2011). Cocaine dynamically regulates heterochromatin and repetitive element unsilencing in nucleus accumbens. Proceedings of the National Academy of Sciences of the United States of America, 108(7), 3035–3040. 21300862 10.1073/pnas.1015483108 PMC3041122

[brb3678-bib-0038] McKay, J. R. , Lynch, K. G. , Coviello, D. , Morrison, R. , Cary, M. S. , Skalina, L. , & Plebani, J. (2010). Randomized trial of continuing care enhancements for cocaine‐dependent patients following initial engagement. Journal of Consulting and Clinical Psychology, 78(1), 111–120. 20099956 10.1037/a0018139 PMC3076098

[brb3678-bib-0039] Mi, H. , Poudel, S. , Muruganujan, A. , Casagrande, J. T. , & Thomas, P. D. (2016). PANTHER version 10: expanded protein families and functions, and analysis tools. Nucleic Acids Research, 44(D1), D336–D342. 26578592 10.1093/nar/gkv1194 PMC4702852

[brb3678-bib-0040] Moszczynska, A. , Flack, A. , Qiu, P. , Muotri, A. R. , & Killinger, B. A. (2015). Neurotoxic methamphetamine doses increase LINE‐1 expression in the neurogenic zones of the adult rat brain. Scientific Reports, 5, 14356. 26463126 10.1038/srep14356 PMC4604469

[brb3678-bib-0041] Muotri, A. R. , Chu, V. T. , Marchetto, M. C. , Deng, W. , Moran, J. V. , & Gage, F. H. (2005). Somatic mosaicism in neuronal precursor cells mediated by L1 retrotransposition. Nature, 435(7044), 903–910. 15959507 10.1038/nature03663

[brb3678-bib-0042] O'Brien, M. S. , & Anthony, J. C. (2005). Risk of becoming cocaine dependent: Epidemiological estimates for the United States, 2000‐2001. Neuropsychopharmacology, 30(5), 1006–1018. Erratum in: Neuropsychopharmacology. 2005 30(8):1588. 15785780 10.1038/sj.npp.1300681

[brb3678-bib-0043] Okudaira, N. , Ishizaka, Y. , & Nishio, H. (2014). Retrotransposition of long interspersed element 1 induced by methamphetamine or cocaine. Journal of Biological Chemistry, 289(37), 25476–25485. 25053411 10.1074/jbc.M114.559419 PMC4162154

[brb3678-bib-0044] Pradhan, L. , Mondal, D. , Chandra, S. , Ali, M. , & Agrawal, K. C. (2008). Molecular analysis of cocaine‐induced endothelial dysfunction: Role of endothelin‐1 and nitric oxide. Cardiovascular Toxicology, 8(4), 161–171. 18813882 10.1007/s12012-008-9025-z

[brb3678-bib-0045] Regier, D. A. , Farmer, M. E. , Rae, D. S. , Locke, B. Z. , Keith, S. J. , Judd, L. L. , & Goodwin, F. K. (1990). Comorbidity of mental disorders with alcohol and other drug abuse. Results from the Epidemiologic Catchment Area (ECA) Study. JAMA, 264(19), 2511–2518. 2232018

[brb3678-bib-0046] Richardson, S. R. , Doucet, A. J. , Kopera, H. C. , Moldovan, J. B. , Garcia‐ Perez, J. L. , & Moran, J. V. (2015). The Influence of LINE‐1 and SINE Retrotransposons on Mammalian Genomes. Microbiology Spectrum, 3(2), MDNA3‐0061‐2014. 10.1128/microbiolspec.MDNA3-0061-2014 PMC4498412 26104698

[brb3678-bib-0047] Rozen, S. , & Skaletsky, H. (2000). Primer3 on the WWW for general users and for biologist programmers In Krawetz S., & Misener S. (Eds.), Bioinformatics methods and protocols (pp. 365–386). Totowa, NJ: Humana Press. 10.1385/1-59259-192-2:365 10547847

[brb3678-bib-0048] Schmidt, H. D. , & Pierce, R. C. (2010). Cocaine‐induced neuroadaptations in glutamate transmission: Potential therapeutic targets for craving and addiction. Annals of the New York Academy of Sciences, 1187, 35–75. Review. 20201846 10.1111/j.1749-6632.2009.05144.x PMC5413205

[brb3678-bib-0049] Scott, A. F. , Schmeckpeper, B. J. , Abdelrazik, M. , Comey, C. T. , O'Hara, B. , Rossiter, J. P. , … Margolet, L. (1987). Origin of the human L1 elements: Proposed progenitor genes deduced from a consensus DNA sequence. Genomics, 1(2), 113–125. 3692483 10.1016/0888-7543(87)90003-6 PMC7135745

[brb3678-bib-0050] Shi, X. , Miller, J. S. , Harper, L. J. , Poole, R. L. , Gould, T. J. , & Unterwald, E. M. (2014). Reactivation of cocaine reward memory engages the Akt/GSK3/mTOR signaling pathway and can be disrupted by GSK3 inhibition. Psychopharmacology (Berl)., 231(16), 3109–3118. 24595501 10.1007/s00213-014-3491-8 PMC4110417

[brb3678-bib-0051] Spronk, D. B. , van Wel, J. H. P. , Ramaekers, J. G. , & Verkes, R. J. (2013). Characterizing the cognitive effects of cocaine: A comprehensive review. Neuroscience and Biobehavioral Reviews, 37, 1838–1859. 23876288 10.1016/j.neubiorev.2013.07.003

[brb3678-bib-0052] Stewart, C. , Kural, D. , Strömberg, M. P. , Walker, J. A. , Konkel, M. K. , Stütz, A. M. , … 1000 Genomes Project (2011). A comprehensive map of mobile element insertion polymorphisms in humans. PLoS Genetics, 7(8), e1002236. 21876680 10.1371/journal.pgen.1002236 PMC3158055

[brb3678-bib-0053] Su, Q. , Cai, Q. , Gerwin, C. , Smith, C. L. , & Sheng, Z. H. (2004). Syntabulin is a microtubule‐associated protein implicated in syntaxin transport in neurons. Nature Cell Biology, 6(10), 941–953. 15459722 10.1038/ncb1169

[brb3678-bib-0054] Substance Abuse and Mental Health Services Administration (SAMHSA) (2014). The national survey on drug use and health report. Retrieved from http://store.samhsa.gov/shin/content//NSDUH14-0904/NSDUH14-0904.pdf 30199191

[brb3678-bib-0055] Sudmant, P. H. , Rausch, T. , Gardner, E. J. , Handsaker, R. E. , Abyzov, A. , Huddleston, J. , … Korbel, J. O. (2015). An integrated map of structural variation in 2,504 human genomes. Nature, 526(7571), 75–81. 26432246 10.1038/nature15394 PMC4617611

[brb3678-bib-0056] Sutton, L. P. , & Caron, M. G. (2015). Essential role of D1R in the regulation of mTOR complex1 signaling induced by cocaine. Neuropharmacology, 99, 610–619. 26314207 10.1016/j.neuropharm.2015.08.024 PMC4703076

[brb3678-bib-0057] Teixeira, C. M. , Masachs, N. , Muhaisen, A. , Bosch, C. , Pérez‐Martínez, J. , Howell, B. , & Soriano, E. (2014). Transient downregulation of Dab1 protein levels during development leads to behavioral and structural deficits: Relevance for psychiatric disorders. Neuropsychopharmacology, 39(3), 556–568. 24030361 10.1038/npp.2013.226 PMC3895234

[brb3678-bib-0058] Tian, W. , Zhao, M. , Li, M. , Song, T. , Zhang, M. , Quan, L. , … Sun, Z. S. (2012). Reversal of cocaine‐conditioned place preference through methyl supplementation in mice: Altering global DNA methylation in the prefrontal cortex. PLoS ONE, 7(3), e33435. 22438930 10.1371/journal.pone.0033435 PMC3306398

[brb3678-bib-0059] Treadwell, S. D. , & Robinson, T. G. (2007). Cocaine use and stroke. Postgraduate Medical Journal, 83(980), 389–394. Review. 17551070 10.1136/pgmj.2006.055970 PMC2600058

[brb3678-bib-0060] Trenz, R. C. , Scherer, M. , Harrell, P. , Zur, J. , Sinha, A. , & Latimer, W. (2012). Early onset of drug and polysubstance use as predictors of injection drug use among adult drug users. Addictive Behaviors, 37(4), 367–372. 22172686 10.1016/j.addbeh.2011.11.011 PMC3288417

[brb3678-bib-0061] Tubio, J. M. , Li, Y. , Ju, Y. S. , Martincorena, I. , Cooke, S. L. , Tojo, M. , … Campbell, P. J. (2014). Mobile DNA in cancer. Extensive transduction of nonrepetitive DNA mediated by L1 retrotransposition in cancer genomes. Science, 345(6196), 1251343. 25082706 10.1126/science.1251343 PMC4380235

[brb3678-bib-0062] Turner, J. R. , Ecke, L. E. , Briand, L. A. , Haydon, P. G. , & Blendy, J. A. (2013). Cocaine‐related behaviors in mice with deficient gliotransmission. Psychopharmacology (Berl)., 226(1), 167–176. 23104263 10.1007/s00213-012-2897-4 PMC3572340

[brb3678-bib-0063] Upton, K. R. , Gerhardt, D. J. , Jesuadian, J. S. , Richardson, S. R. , Sánchez‐Luque, F. J. , Bodea, G. O. , … Faulkner, G. J. (2015). Ubiquitous L1 mosaicism in hippocampal neurons. Cell, 161(2), 228–239. 25860606 10.1016/j.cell.2015.03.026 PMC4398972

[brb3678-bib-0064] White, T. B. , McCoy, A. M. , Streva, V. A. , Fenrich, J. , & Deininger, P. L. (2014). A droplet digital PCR detection method for rare L1 insertions in tumors. Mobile DNA, 5(1), 30. 25598847 10.1186/s13100-014-0030-4 PMC4297411

[brb3678-bib-0065] Winstanley, C. A. , LaPlant, Q. , Theobald, D. E. , Green, T. A. , Bachtell, R. K. , Perrotti, L. I. , … Nestler, E. J. (2007). DeltaFosB induction in orbitofrontal cortex mediates tolerance to cocaine‐induced cognitive dysfunction. Journal of Neuroscience, 27(39), 10497–10507. 17898221 10.1523/JNEUROSCI.2566-07.2007 PMC6673166

[brb3678-bib-0066] Yao, H. , Duan, M. , & Buch, S. (2011). Cocaine‐mediated induction of platelet‐derived growth factor: Implication for increased vascular permeability. Blood, 117(8), 2538–2547. Erratum in: Blood. 2015 Jun 11;125(24):3823. 21148086 10.1182/blood-2010-10-313593 PMC3062415

