# Supplementary figures and images for: Reading LINEs within the cocaine addicted brain
Source: Brain Behav. 2017 Apr 6;7(5):e00678. doi: 10.1002/brb3.678 (PMC5434184; doi:10.1002/brb3.678)

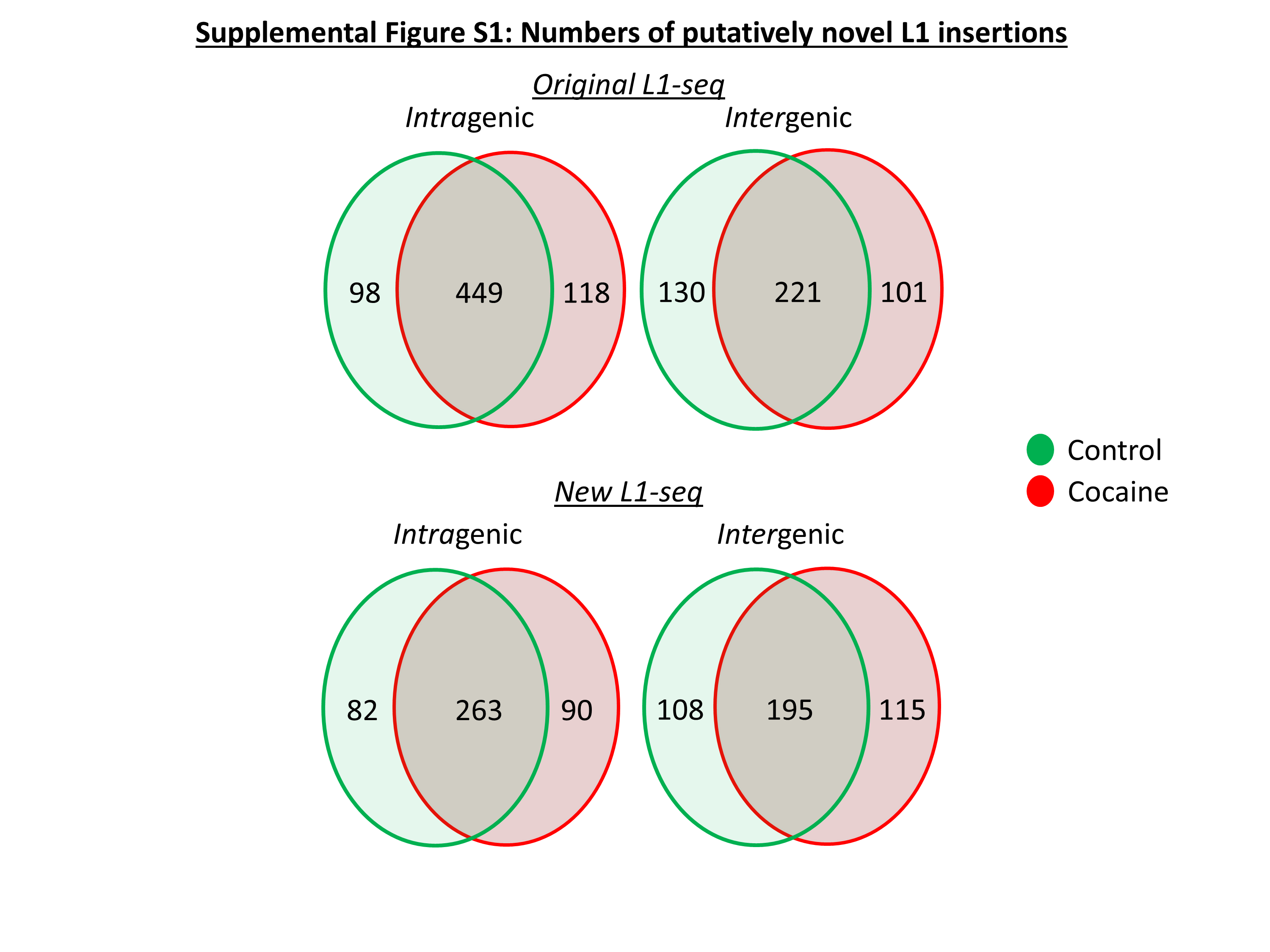

Supplement: Supplementary file 1 [file BRB3-7-e00678-s001.tiff]

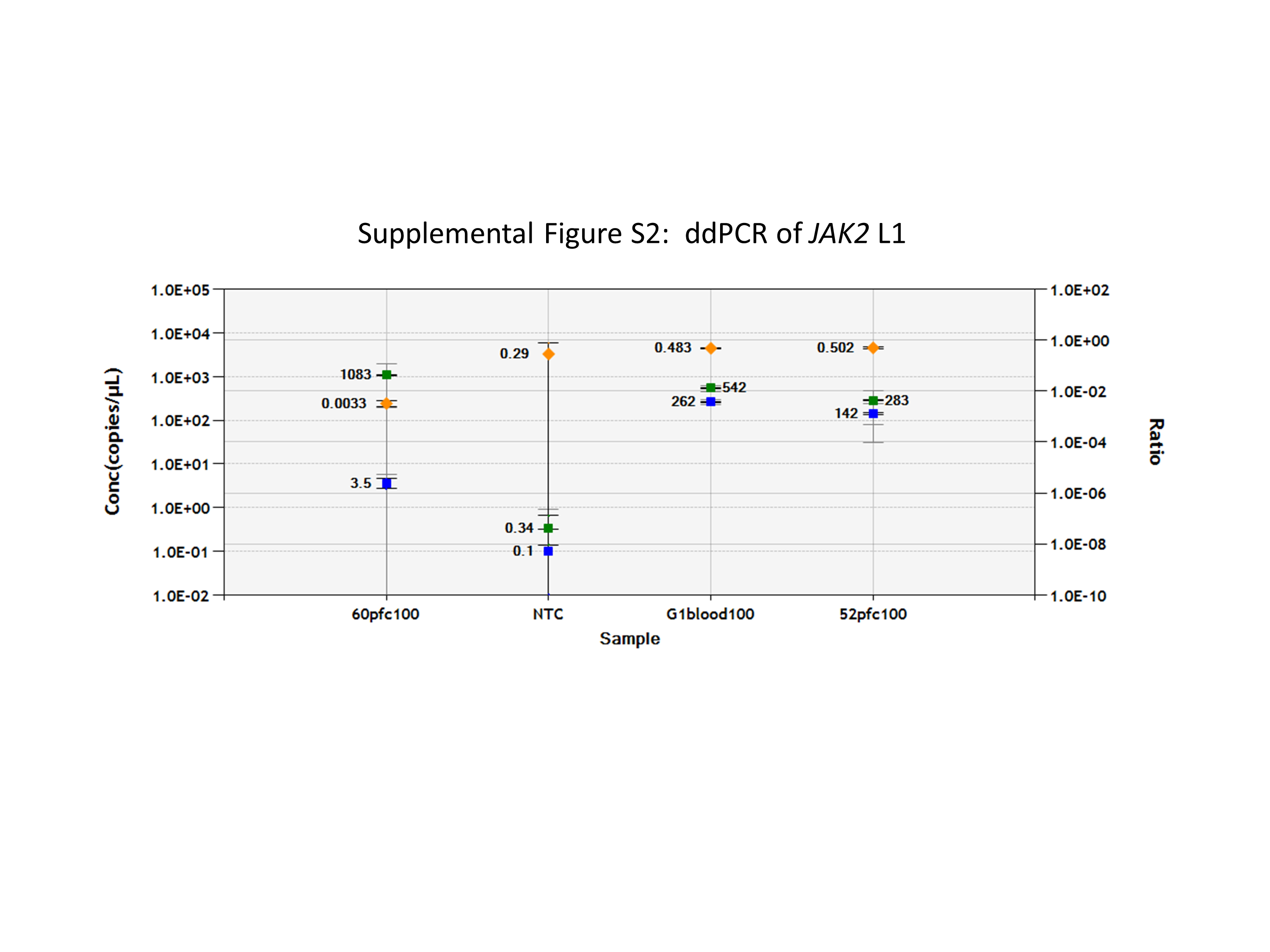

Supplement: Supplementary file 2 [file BRB3-7-e00678-s002.tiff]
