# Supplementary material for: Reading LINEs within the cocaine addicted brain
Source: Brain Behav. 2017 Apr 6;7(5):e00678. doi: 10.1002/brb3.678 (PMC5434184; doi:10.1002/brb3.678)
Supplement: Supplementary file 4 [file BRB3-7-e00678-s004.docx]

DAVID (32) employs a hypergeometric distribution to calculate fold enrichment and *p*-values according to the equations:


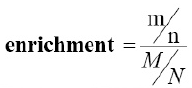


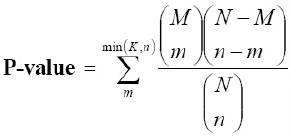


Where: N=number of all genes, M=number of genes in a given pathway, n=list of genes input into the analyses, and m=number of input genes belonging to the pathway.

PANTHER (33) employs a binomial distribution to calculate fold enrichment and *p*-values according to the equations:

p(C)=n(C)/N;

Where: n(C) is the number of genes mapped to category C, and N is the total number of genes in the reference set.

The *p*-value is calculated according to the equation:

$$p-value=\sum\left( \begin{aligned} K \\ k \end{aligned} \right){p(c)}^{k}{(1-p\left( c \right))}^{K-k}$$

Where: the sum runs from k(C) to K in the case of over-representation (i.e. when the number of observed genes k(C) is greater than expected p(C)*K under the NULL hypothesis), and 0 to k(C), in the case of under-representation (i.e. when k(C) is smaller than p(C)*K).
