# Supplementary material for: Reading LINEs within the cocaine addicted brain
Source: Brain Behav. 2017 Apr 6;7(5):e00678. doi: 10.1002/brb3.678 (PMC5434184; doi:10.1002/brb3.678)
Supplement: Supplementary file 8 [file BRB3-7-e00678-s008.pdf]

SYBU-Empty Allele

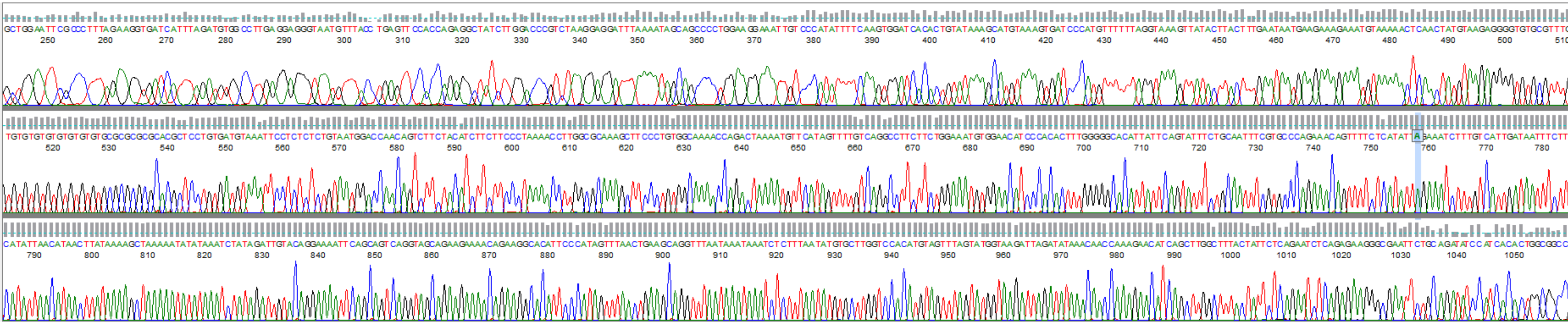

SYBU-L1 Allele

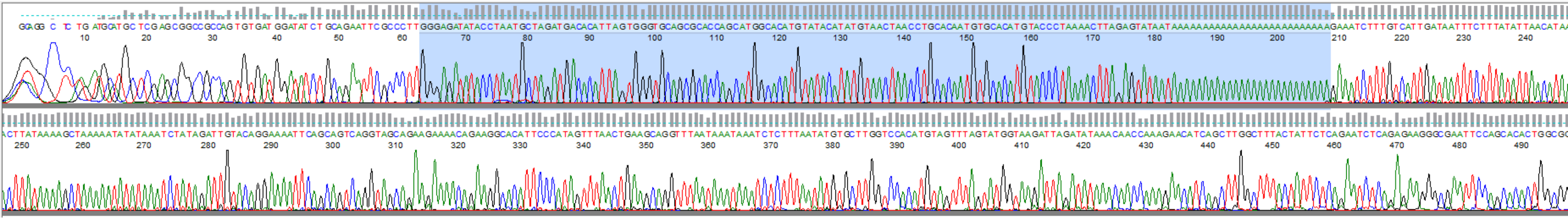

SYBU L1 insertion (highlighted in bottom panel) is at position 759-A (highlighted in top panel) adjacent to a TTTC (reverse-complement) sequence.

TET2-L1 Allele

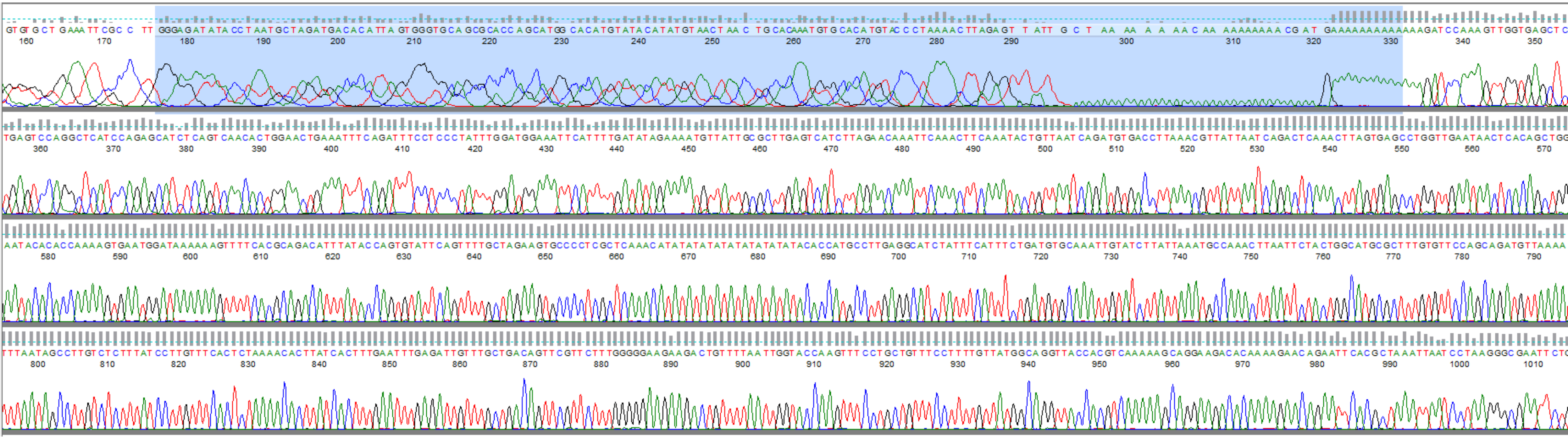

TET2 L1 (highlighted) inserted adjacent to a TCTT (reverse-complement) sequence.

WBSCR17-Empty Allele

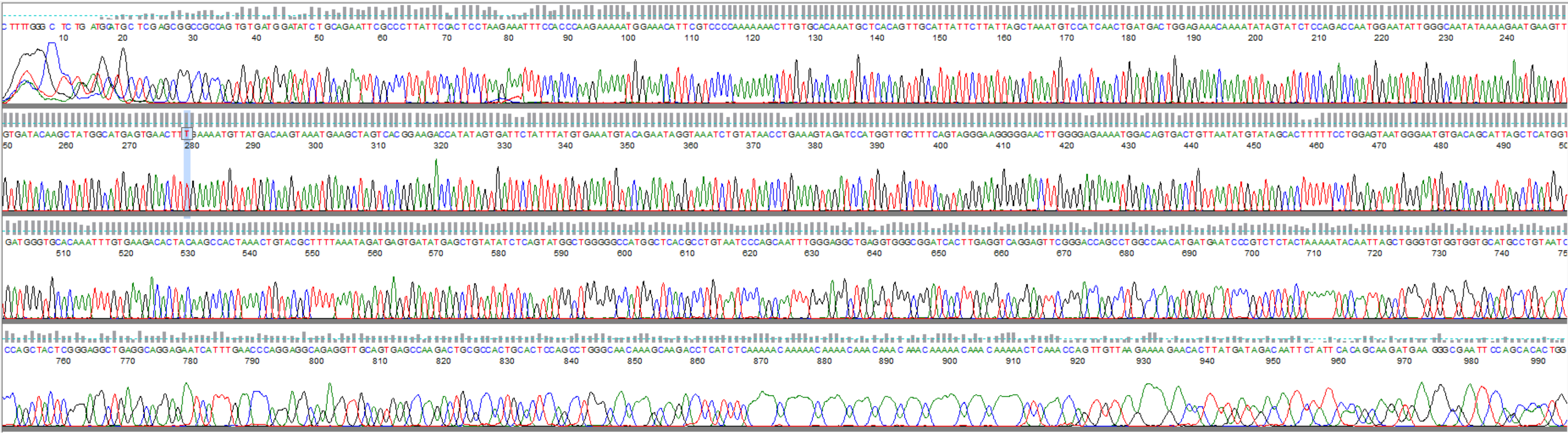

WBSCR17-L1 Allele

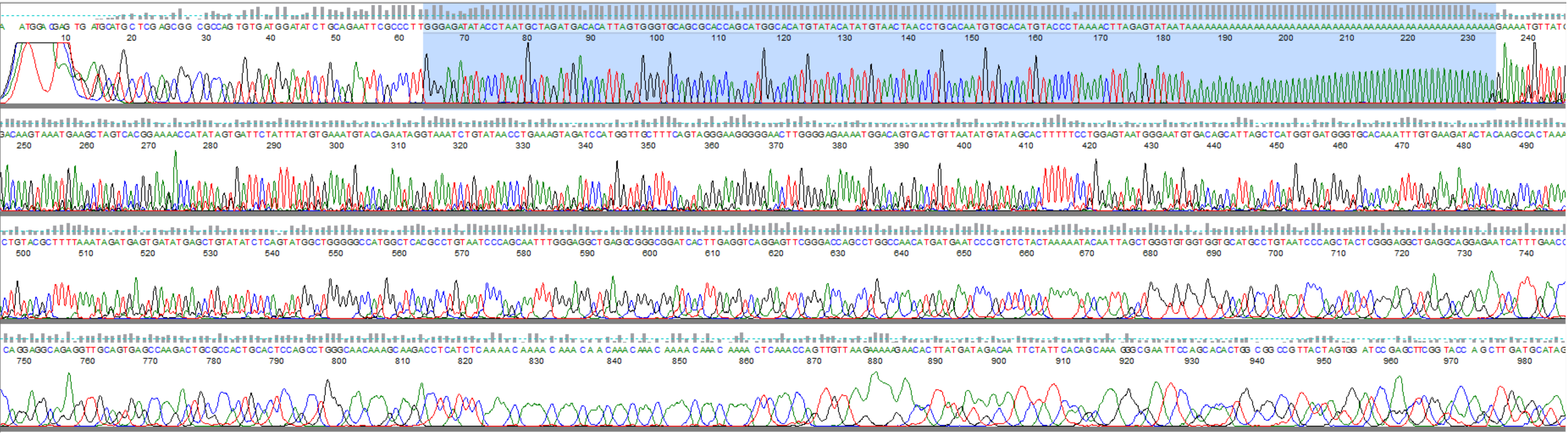

WBSCR17 L1 insertion (highlighted in bottom panel) is at position 279-T (highlighted in top panel) adjacent to a TTTC (reverse-complement) sequence.

DAB1-Empty Allele

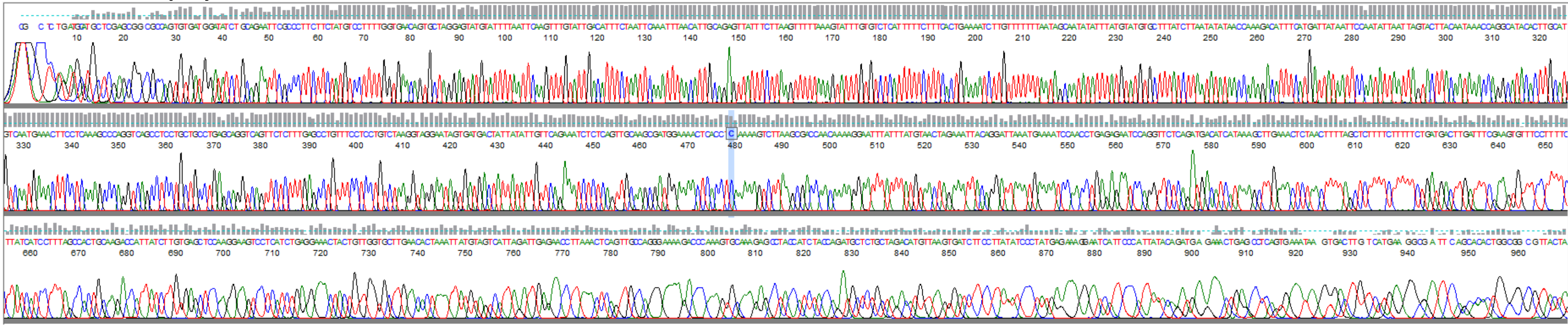

DAB1-L1 Allele

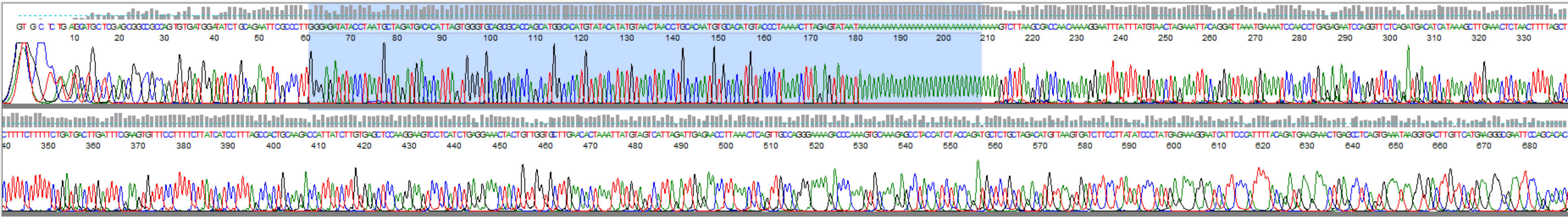

DAB1 L1 insertion (highlighted in bottom panel) is at position 479-C (highlighted in top panel) adjacent to a TTTT (reverse-complement) sequence.

KLHL1-Empty Allele

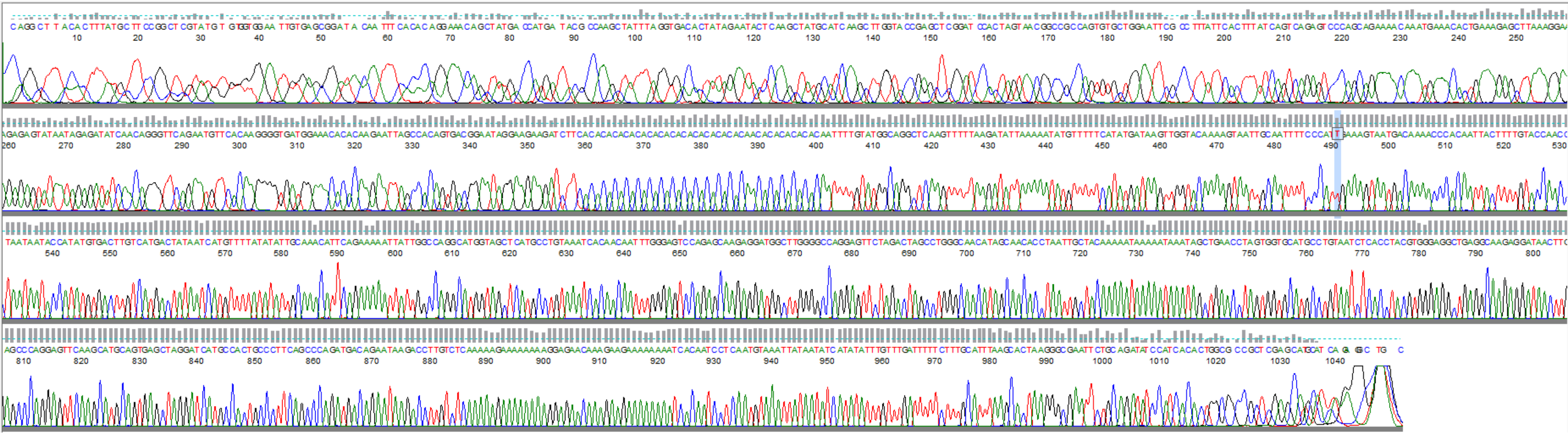

KLHL1-L1 Allele

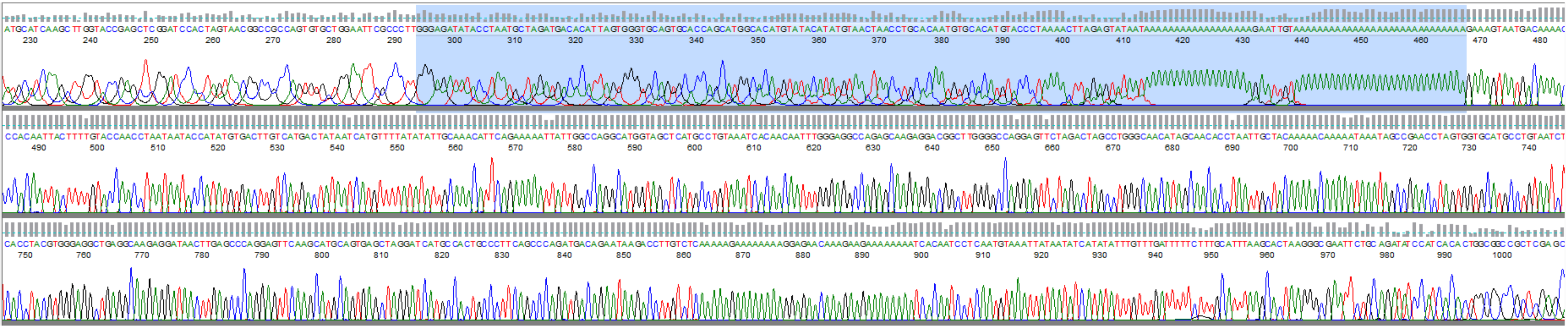

KLHL1 L1 insertion (highlighted in bottom panel) is at position 491-T (highlighted in top panel) adjacent to a TTTC (reverse-complement) sequence.

TBCK-Empty Allele

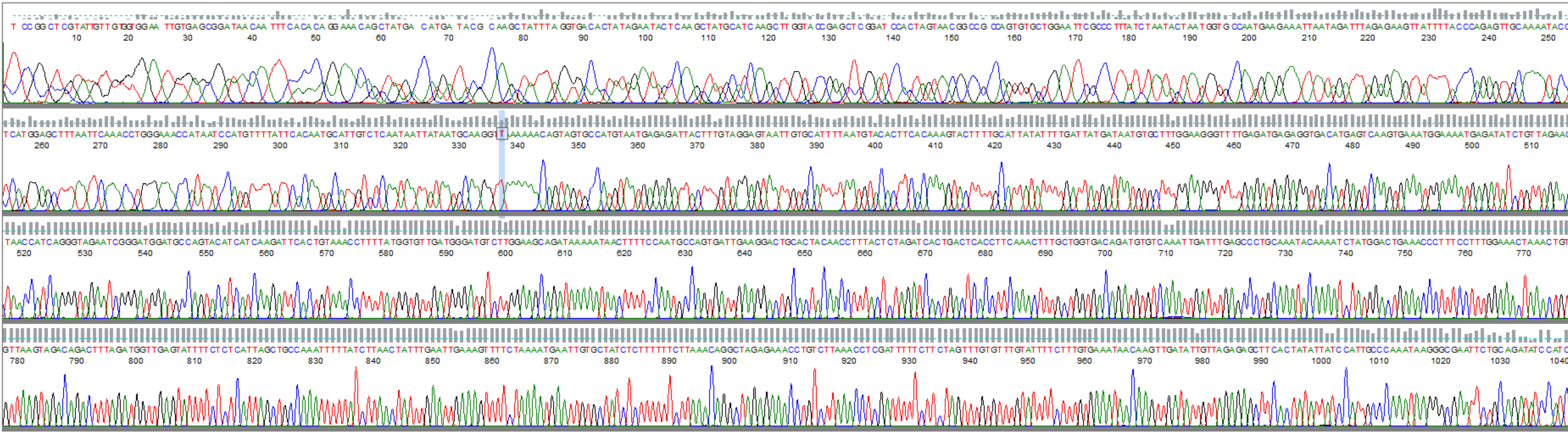

TBCK-L1 Allele

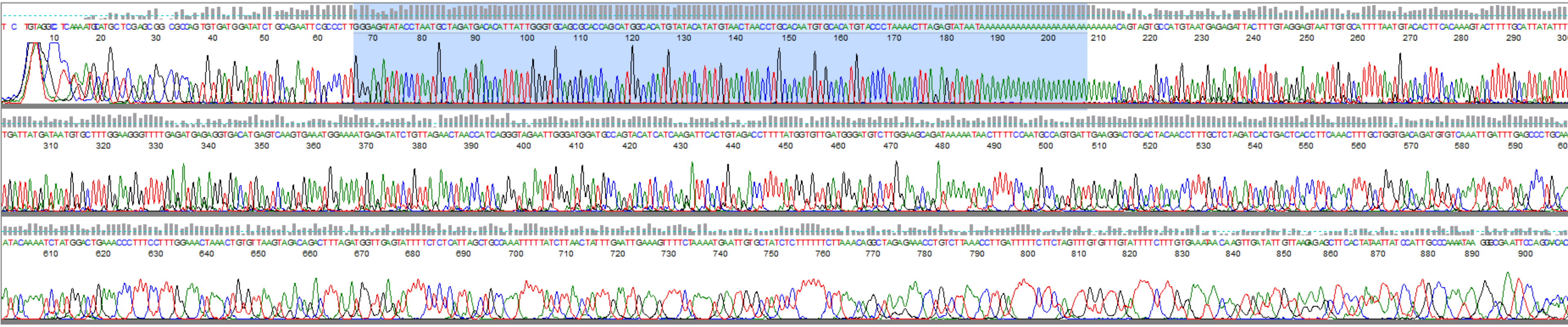

TBCK L1 insertion (highlighted in bottom panel) is at position 337-T (highlighted in top panel) adjacent to a TTTT (reverse-complement) sequence.

ATXN1-L1 Allele

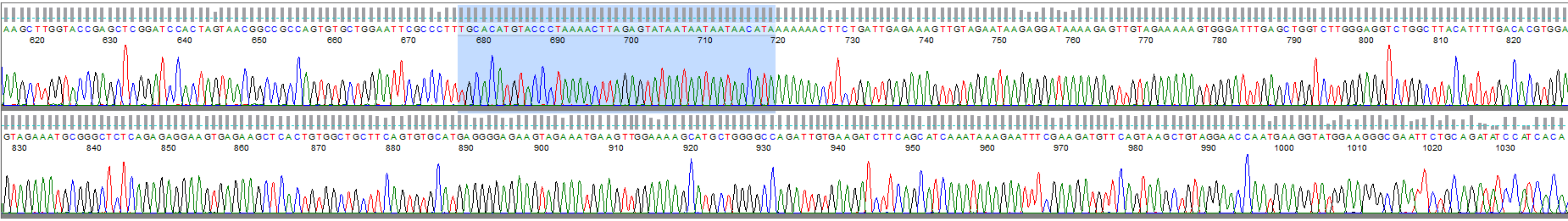

ATXN1 L1 insertion (highlighted) is adjacent to a TTTT (reverse-complement) sequence.

CTCF-L1 Allele

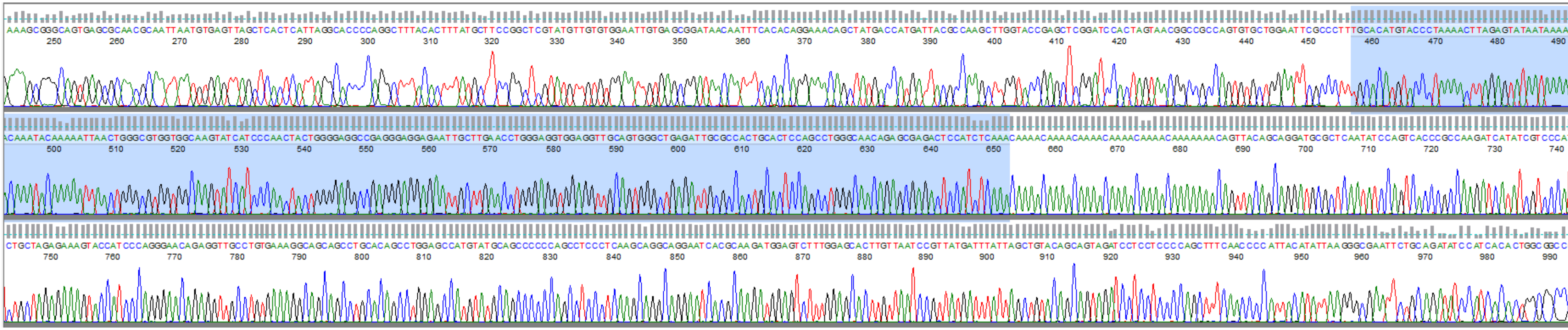

CTCF L1 insertion (highlighted) includes a 3' transduction and inserted within a simple CAAAA repeat adjacent to a TTTG (reverse-complement) sequence.

PTHR2-L1 Allele

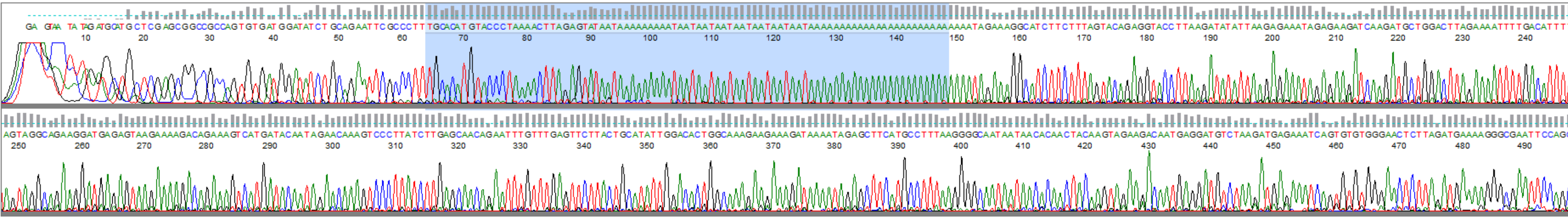

PTHR2 L1 insertion (highlighted) inserted adjacent to a TTTT (reverse-complement) sequence.

DDX58-L1 Allele

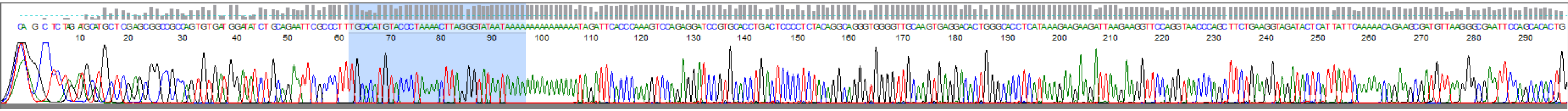

DDX58 L1 insertion (highlighted) inserted adjacent to a TTTT (reverse-complement) sequence.

MACROD2-L1 Allele

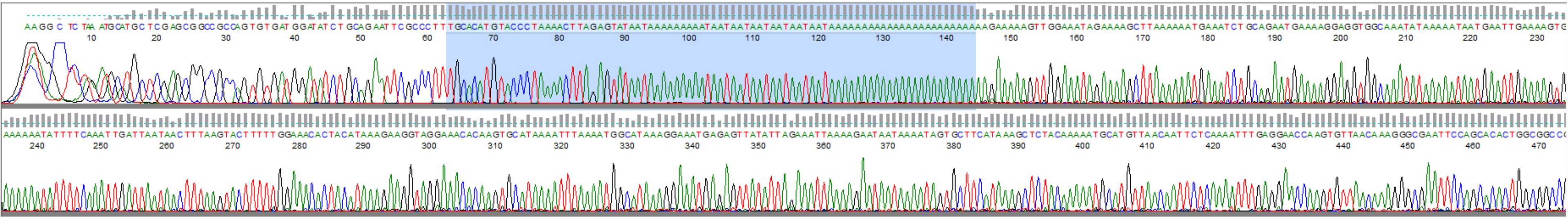

MACROD2 L1 insertion (highlighted) is adjacent to a TCTT (reverse-complement) sequence.

DACH2-L1 Allele

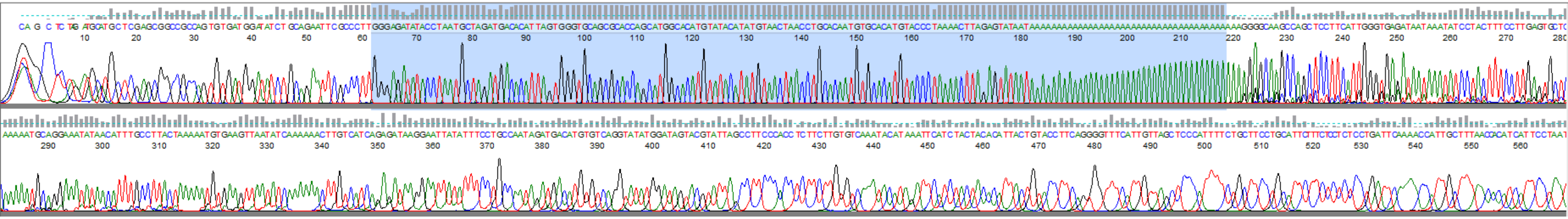

DACH2 L1 insertion (highlighted) is adjacent to a CTTT (reverse-complement) sequence.
