# Supplementary material for: Reading LINEs within the cocaine addicted brain
Source: Brain Behav. 2017 Apr 6;7(5):e00678. doi: 10.1002/brb3.678 (PMC5434184; doi:10.1002/brb3.678)
Supplement: Supplementary file 9 [file BRB3-7-e00678-s009.docx]

**Supplemental Figure Legends**

**Supplemental Figure S1:** Venn diagrams of *intra*genic and *inter*genic putatively novel L1 insertions in control and cocaine mPFC samples detected using ‘original’ and ‘new’ L1-seq bioinformatics.

**Supplemental Figure S2:** **ddPCR allele frequency of the L1 in *JAK2* .**  Graph showing the absolute copy numbers of *JAK2*-L1 (blue squares) and *RPPH1* (green squares) genes, as well as the ratios (*JAK2*-L1:*RPPH1*, orange diamonds), detected in 100 ng of XmnI-digested gDNA from mPFC (“60pfc100”) of CA individual 60, who did not have the *JAK2*-L1, or from 100 ng of XmnI-digested gDNA from mPFC (“52pfc100”) of CA individual 52, who did have the *JAK2*-L1. Blood gDNA from an individual (“G1blood100”) in our repository samples of cocaine addicts was also positive for the *JAK2*-L1. NTC is the no template control.
